# Molecular mechanisms underlying the actions of arachidonic acid-derived prostaglandins on peripheral nociception

**DOI:** 10.1186/s12974-020-1703-1

**Published:** 2020-01-22

**Authors:** Yongwoo Jang, Minseok Kim, Sun Wook Hwang

**Affiliations:** 1 Department of Psychiatry and Program in Neuroscience, McLean Hospital, Harvard Medical School, Belmont, MA 02478 USA; 2 Department of Biomedical Engineering, Hanyang University, Seoul, 04763 South Korea; 3 Department of Biomedical Sciences, Korea University, Seoul, 02841 South Korea; 4 Department of Physiology, College of Medicine, Korea University, Seoul, 02841 South Korea

**Keywords:** Inflammation, Prostaglandin, Pain, Signal transduction, DRG neuron

## Abstract

Arachidonic acid-derived prostaglandins not only contribute to the development of inflammation as intercellular pro-inflammatory mediators, but also promote the excitability of the peripheral somatosensory system, contributing to pain exacerbation. Peripheral tissues undergo many forms of diseases that are frequently accompanied by inflammation. The somatosensory nerves innervating the inflamed areas experience heightened excitability and generate and transmit pain signals. Extensive studies have been carried out to elucidate how prostaglandins play their roles for such signaling at the cellular and molecular levels. Here, we briefly summarize the roles of arachidonic acid-derived prostaglandins, focusing on four prostaglandins and one thromboxane, particularly in terms of their actions on afferent nociceptors. We discuss the biosynthesis of the prostaglandins, their specific action sites, the pathological alteration of the expression levels of related proteins, the neuronal outcomes of receptor stimulation, their correlation with behavioral nociception, and the pharmacological efficacy of their regulators. This overview will help to a better understanding of the pathological roles that prostaglandins play in the somatosensory system and to a finding of critical molecular contributors to normalizing pain.

## Introduction

**Fig. 1:**
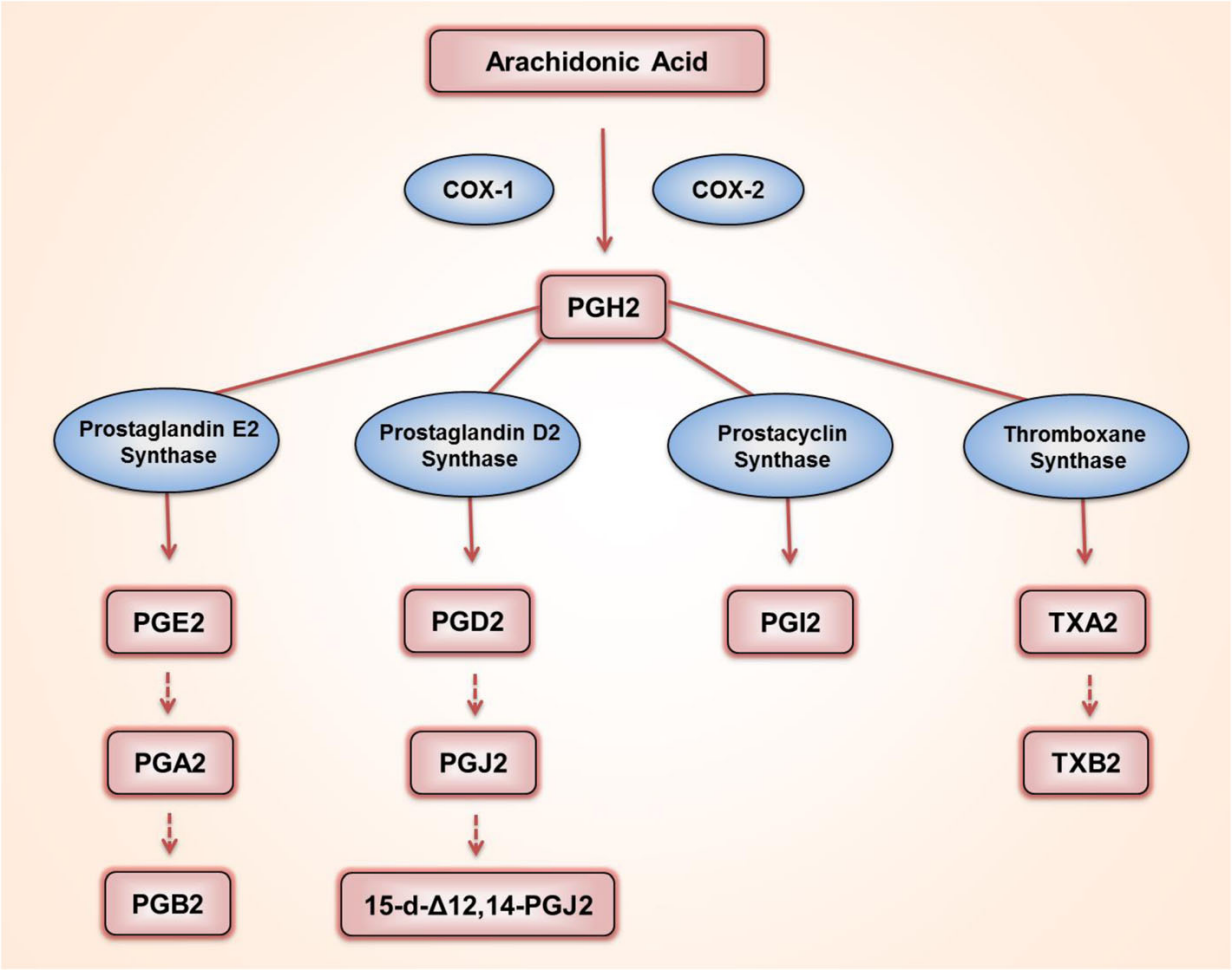
Biosynthetic pathways of arachidonic acid-mediated prostaglandins (PGs). PGH_2_ is generated from arachidonic acid by enzymatic reactions of COX-1 or COX-2, and is further metabolized into PGE_2_, PGD_2_, PGI_2_, or TXA_2_ by specific synthases. When dehydrated, PGE_2_ is converted into PGA_2_ and PGB_2_, and PGD_2_ is converted into PGJ_2_. PGJ_2_ can further be isomerized into 15-deoxy-Δ12,14-PGJ_2_ (15d-PGJ_2_). TXA_2_ is rapidly hydrolyzed into TXB_2_

Polyunsaturated fatty acids are oxygenated via cellular enzymatic processes [1]. Cyclooxygenase (COX, also known as prostaglandin G/H synthase {PTGS}), epoxygenase, and lipoxygenase catalyze those reactions [1]. The resulting oxygenated metabolites, termed eicosanoids, function as crucial bioactive lipids. Near or inside the peripheral somatosensory system, these eicosanoids play diverse roles in the pro-inflammatory, anti-inflammatory, and resolving phases of injury. During those phases, secreted eicosanoids often greatly alter the functions of neuronal components [2]. Arachidonic acid is a C20 polyunsaturated ω-6 fatty acid. Among the arachidonic acid-derived eicosanoids, prostaglandin G2 (PGG_2_) and subsequently H_2_ (PGH_2_) are first generated by the actions of COX, and can then be further metabolized into PGE_2_, PGD_2_, PGI_2_, and TXA_2_ by a corresponding prostaglandin synthase (Fig. 1) [3]. PGA_2_ and PGJ_2_ are formed by the dehydration of PGE_2_ and D_2_, respectively. In a paracrine or autocrine manner, most of these PGs preferentially recognize one or more receptors coupled to G proteins expressed on the cell surface. That interaction then initiates intracellular signal transductions, including cyclic adenosine monophosphate (cAMP)- and calcium ion (Ca^2+^)-induced cascades, which can occur, among other places, in the neuronal components constituting somatosensory ganglia experiencing inflammation in or around themselves [4]. Many studies have revealed that the receptor-specific actions of PGs mostly heighten neuronal excitability, which can cause pro-nociceptive outcomes. In this review, we focus on the contribution of each specific PG action to pain signaling and construct systemic information to describe the molecular mechanisms that underlie their actions on the neurons of the somatosensory ganglia.

## COX in the peripheral somatosensory system

COX, the key element in the biosynthetic pathway of PGs, catalyzes the following serial reactions: the cyclooxygenation of arachidonic acid to PGG_2_ and a subsequent peroxidation that reduces PGG_2_ to PGH_2_ [3] (Fig. 1). In humans, COX has two isoforms, COX-1 and COX-2, and their expressions and functions are separately regulated in various tissues [5]. When inflamed, tissues and recruited inflammatory cells increase PG production and secretion, and the secreted PGs can stimulate the innervating neurons in a paracrine fashion [6]. In addition, the neuron itself also has the potential to generate PGs because it also expresses COX. In the dorsal root ganglia (DRG), which are collections of cell bodies of somatosensory neurons, and in the spinal cord, where DRG neurons form their first synapses, COX isoform expression that depends on physiological and inflammatory conditions has been investigated as follows.

### COX expression in the spinal cord and somatosensory neurons

Spinal cord expression of COX-1 and COX-2 was first examined in 1996. Reverse transcription-polymerase chain reaction (RT-PCR) analyses showed that both of the *Ptgs* transcripts encoding COX-1 and -2 proteins were constitutively expressed in the rat spinal cord and that *Ptgs2* was predominant [7]. Beiche et al. further demonstrated that peripheral inflammation induced by hind paw injection of complete Freund’s adjuvant (CFA) up-regulated the lumbar spinal expression of *Ptgs2* but not *Ptgs1*, which indicates that COX-2 is more important than COX-1 in that pathological state. Such spinal expressions have repeatedly been confirmed in multiple animal models [6, 8–12]. For example, an intraperitoneal injection of the endotoxin lipopolysaccharide (LPS) (1 mg/kg) in mice significantly elevated the levels of *Ptgs2* mRNA and COX-2 protein in the spinal cord, with no change in the levels of *Ptgs1* or COX-1 protein [13]. Immunohistochemistry has later been employed to obtain lamina-specific information regarding COX-1 and -2 expression, because the synaptic transmission of nociceptive signals from nociceptor DRG neurons in response to noxious peripheral stimuli occurs in the superficial dorsal horn (laminae I and II). The result showed that the a normal spinal cord expressed the COX-1 isoform throughout the whole gray matter area, whereas COX-2 expression was relatively intense in laminae I and II, as well as around the central canal (lamina X) [14]. A comparison study using transgenic mice (wild-type mice and heterozygous and homozygous knockouts for the *Ptgs1* and *Ptgs2* genes) confirmed the spinal expression of both enzymes [15]. Collectively, therefore, the COXs are commonly expressed in the spinal cord, and peripheral inflammation can preferentially lead to an increase in COX-2 expression (Table 1).

**Table 1:** Expressions of COX-1 and COX-2 in dorsal root ganglia (DRG) and spinal cord

DRG and/or spinal cord	COX isoforms	Animal models	Expression	References
lumbar spinal cord	COX-2 mRNA	Freund’s adjuvant-induced rat	Increase	[7]
gray matter of the spinal cord	COX-1 protein	Normal rat	Detection	[14]
superficial dorsal horn of the spinal cord (laminae I and II) Around the central canal (lamina X)	COX-2 protein	Normal rat	Detection	[14]
small to medium sized (< 1000 μm^2^) DRG	COX-1 protein	Normal rat	Detection	[14, 16]
DRG	COX-2 protein	Normal rat	No detection	[14, 16]
spinal cord	COX-1, COX-2 protein	Kaolin and carrageenan-induced arthritis rat	No change in COX-1, increase in COX-2	[8]
spinal cord	COX-1 mRNA	COX-2-deficient mice	Increase	[15]
spinal cord	COX-2 mRNA	COX-1-deficient mice	No change	[15]
part of COX-1-positive DRG neurons	COX-1 protein	Freund’s adjuvant-injected rat	No change	[16]
DRG	COX-2 protein	Freund’s adjuvant-injected rat	No detection	[16]
spinal cord	COX-2 protein	Freund’s adjuvant-injected rat	Increase	[6]
L4 and L5 DRG	COX-1 protein	COX-1/COX-2-deficient mice	No detection in COX-1	[17]
L4 and L5 DRG	COX-2 protein	COX-1/COX-2-deficient mice	No detection	[17]
L4 and L5 DRG	COX-1, COX-2 protein	Normal mouse	No detection in COX-2	[17]
L4 – L6 DRG and spinal cord,	COX-1, COX-2 protein	Normal rat	Detection	[10]
spinal cord	COX-2 mRNA / protein	Formalin-induced inflamed rat	Increase in mRNA, no change in protein	[9]
L4 and L5 DRG	COX-1 mRNA	Collagen-induced arthritis mouse	No change	[17]
L4 and L5 DRG	COX-2 mRNA	Collagen-induced arthritis mouse	No detection	[17]
spinal cord	COX-1 mRNA / Protein	LPS-induced inflamed mouse	No change	[13]
spinal cord	COX-2 mRNA / Protein	LPS-induced inflamed mouse	Increase	[13]
spinal cord	COX-2 mRNA / protein	LPS-induced inflamed mouse	increase	[18]
DRG and spinal cord	COX-1, COX-2 mRNA	Carrageenen-induced inflamed mouse	No change in COX-1, Increaser in COX-2	[19]
L4 DRG	COX-2 mRNA / protein	Freund’s adjuvant-injected rat	Increase	[20]
TRPV1-positive cells in L5 DRG	COX-1, COX-2 protein	IL-1β- or carrageenan-induced rat	Increase	[21]

DRG neurons are presynaptic components in the spinal dorsal horn and are anatomically and functionally classified into four subpopulations: C-nociceptors, Aδ-nociceptors, Aβ touch fibers, and Aα-proprioceptors. The unmyelinated C- and thinly myelinated Aδ-fibers have relatively small-to-medium-sized cell bodies and axons and are essential for pain perception because they transduce potentially harmful stimuli such as noxious ranges of temperatures, damaging stretches, and painful substances into electrical signals [22, 23]. The thickly myelinated A-fibers (Aα- and Aβ- fibers) have relatively large somas and axons and are responsible for the transduction of innocuous mechanical stimuli [22, 23].

Comparisons of such subpopulations have reported differential COX expression levels. The COX-1 isoform has been detected in the cytosolic compartment and axonal processes of small-to-medium sized rat DRG neurons, but COX-2 was not expressed in those neurons [14, 16]. The same research group further detected COX-1 in 65% of calcitonin gene-related peptide (CGRP)-positive and 70% of isolectin B4 (IB4)-positive DRG neurons, suggesting that the majority of both peptidergic and non-peptidergic small-diameter nociceptors express COX-1 [16]. Dou and colleagues confirmed the presence of COX-1 and absence of COX-2 in mouse lumbar (L4 and L5) DRG and further revealed the presence of a new variant of COX-1 containing intron-1 (referred to as COX-3, which is not expressed in humans) in those lumbar DRG [17]. Regarding COX-2 expression, they used a collagen-induced arthritis model and found that *Ptgs2* mRNA was increased in inflamed hind paw skin and the lumbar spinal cord, but remained absent in the L4 and L5 DRG [17]. Interestingly, recent studies have suggested that certain pathological states can cause COX-2 expression in DRG neurons. For example, carrageenan-induced peripheral inflammation elevated the *Ptgs2* mRNA level in DRG neurons [19]. L4 periganglionic inflammation caused by the injection of CFA robustly induced COX-2 expression in the L4 DRG [20]. Araldi and colleagues also showed that epidermal administration of interleukin-1β (IL-1β) or carrageenan to rat hind paws elevated COX-1 and COX-2 levels in the nociceptive ion channel transient receptor potential vanilloid subtype 1 (TRPV1)-positive DRG nociceptors in charge of the injected dermatome [21]. Therefore, PGs can be produced in nociceptor afferents in addition to their innervated areas when inflamed and possibly affect neuronal functions in autocrine and/or paracrine manners. According to this collective information on COX expression, both the pro-inflammatory enzymes COX-1 and COX-2 could be involved in the nociception that is exerted by peripheral somatosensory components, and COX-2 expression can be up-regulated upon inflammation.

### COX implication in pain from genetic approaches

In pain behavioral tests using transgenic animals, heat nociception was reduced in *Ptgs1*-null mice, and chemical nociception in response to acetic acid was blunted in *Ptgs1*-deficient heterozygotes, *Ptgs2*-deficient female heterozygotes, and *Ptgs1*-null mice [15]. Despite the difficulties in interpreting these behavioral parameters because of the spinal increase in COX-1 seen in *Ptgs2* knockouts, which possibly resulted from a compensatory mechanism, those authors suggested that both of the COXs serve a role in pain development [15]. However, to further specify the peripheral contribution of individual COXs in the peripheral pain pathway, more systematic approaches might be required, such as using conditional deletions of *Ptgs* in the spinal cord or DRG neurons. In fact, a similar concept was recently attempted. Animals were generated to have tissue-specific knockdowns of *Ptgs1* or *Ptgs2* by directly injecting antisense oligodeoxynucleotides (ODNs) in the L5 DRG, and both of the knockdown treatments prevented the hind paw hyperalgesia induced by IL-1β injection [21]. Therefore, the ascending neural pathway for pain signaling composed of the DRG and spinal cord expresses COX-1 and COX-2, and those two enzymes appear to contribute to nociceptive transduction.

### Pharmacological evidence for COX actions in nociceptors

Pharmacological studies have shown that COX-mediated peripheral nociceptive mechanisms contribute to pain in various pathological models (Table 2 summarizes the COX selectivity of various pharmacological agents). Those can be subcategorized into three types of approaches: nociceptor-specific measures, peripherally localized treatment, and peripherally localized stimulation.

**Table 2:** COX inhibitors used in the experiments mentioned in this review

Type	Name	Inhibitors	Notes (Molecular or cellular outcomes except reductions in PGs or pain)	References
Selective COX-1 inhibitors	COX-1	Valeryl salicylate	↓ IL-1β-induced hyperalgesia	[21]
		SC-560	↓VEGF-induced growth cone formation	[9, 10, 24, 25]
Selective COX-2 inhibitors	COX-2	Celecoxib	↓substance P,↓TTX-R I_Na_, ↓CGRP, ↓P2X3 expression	[9, 26–28]
		Lumiracoxib		[13]
		Meloxicam	↓neurogenesis	[29, 30]
		Nimesulide	↓neurogenesis, ↓substance P, ↓PKCε translocation	[26, 30]
		NS-398	↓BDNF	[6, 25, 31–35]
		Rofecoxib		[20]
		SC-236	↓ IL-1β-induced hyperalgesia	[10, 21]
		SC-58125	↓firing magnitude of C-nociceptors	[10, 14, 29]
Nonselective COX inhibitors	COX-1 and COX-2	Diclofenac	↓substance P	[26]
		Ibuprofen	↓TTX-R expression, ↓P2X3 expression	[10, 26, 28, 32, 36]
		Indomethacin	↓BK-mediated CGRP release, ↓firing magnitude of C-nociceptors, ↓ IL-1β-induced hyperalgesia, ↓TNFα-sensitized neuronal response to capsaicin, ↓VEGF-induced growth cone formation	[14, 21, 25, 29, 37–42]
		Ketorolac		[36]
		Paracetamol	↓PKCε translocation	[26]
		Piroxicam		[43]

#### Studies using nociceptor specific measures

In an earlier study on the wind-up of a spinal nociceptive reflex in rats, systemic administration of the non-selective COX inhibitor indomethacin or the selective COX-2 inhibitor SC-58125 reduced the firing magnitude of C-nociceptors in a dose-dependent manner when evoked by electrical stimulation at a frequency of 0.5–0.8 Hz [14]. In contrast, the intrathecal administration of the COX inhibitors indomethacin and meclofenamic acid failed to suppress the responses of spinal dorsal horn neurons to noxious mechanical stimulation at the ankle or knee joint [14].

CFA-induced periganglionic inflammation led to mechanical and thermal hyperalgesia of the relevant hind paw with increased COX-2 expression in DRG neurons, which was blunted by the subcutaneous administration of rofecoxib, a COX-2-specific inhibitor (1 mg/kg) [20].

Substance P (SP) is a peptidergic neurotransmitter released from a subset of C-fibers in the DRG [44]. Its release from the central ending in the dorsal horn strengthens the synaptic transmission of pain signals and its release from the peripheral terminal causes neurogenic inflammation [44]. In vitro stimulation of cultured DRG neurons with an inflammatory soup containing potassium chloride, thrombin, bradykinin (BK), and endothelin-1 led to increased neuronal transcription of preprotachykinin, which is an SP precursor, and increased SP release [26]. The COX inhibitors nimesulide and diclofenac and COX-2 inhibitor celecoxib all deterred those SP induction processes [26]. Moreover, treating cultured DRG neurons with the COX inhibitors nimesulide and paracetamol suppressed the translocation of epsilon type protein kinase C (PKCε) to the plasma membrane by thrombin and BK, which is mentioned below as an essential axis for PGE_2_ downstream signaling [26]. The same study confirmed that both baseline and inflammatory releases of PGE_2_ from DRG neurons were reduced after treatment with several COX inhibitors (nimesulide, celecoxib, diclofenac, ibuprofen, and paracetamol) [26].

Interestingly, resveratrol, a natural polyphenol (2 mg/kg), exhibited an anti-nociceptive effect in carrageenan-evoked hyperalgesia in rats when administered intraperitoneally, presumably by suppressing COX-2 expression in the DRG and spinal cord [19].

#### Studies using localized pharmacological treatments

The effect of local administrations has been examined using the partial sciatic nerve ligation (pSNL) model in rats [29]. In addition to indomethacin, the COX-2 inhibitors meloxicam and SC-58125 showed analgesic efficacy when subcutaneously injected into injured hind paws. The analgesia was restricted to the injected paws, implying that the mechanism may probably involve altered nociceptor function. Again in rats aged 18 months after pSNL, sciatic nerve perineural injection of NS-398 (a COX-2 inhibitor) also relieved chronic neuropathic pain [45]. In another study, the direct injection of a COX inhibitor (indomethacin, valeryl salicylate, or SC-236) into the L5-dominated peripheral field has commonly been shown to alleviate IL-1β-induced hyperalgesia in the hind paws of rats [21].

#### Studies using localized stimulation

Subcutaneous injection of the isoprostanes 8-iso PGE_2_ and 8-iso PGF_2α_ has been shown to acutely lower the von Frey mechanical threshold, presumably through nociceptor sensitization. Those allodynic responses were at least partly rescued by ketorolac (1 and 10 mg/kg) and ibuprofen treatments (30 mg/kg) and thus those authors suggested that not only isoprostane-specific receptor molecule-mediated sensitization, but local prostaglandin production were also responsible for the acute sensitization [36]. Overall, consistent with the results from transgenic studies of COXs, the results from studies of their pharmacological inhibition including cases specifically targeting the peripheral somatosensory system indicate that they contribute to pathological nociception.

## PGE_2_ and PGD_2_

Which PGs most actively contribute to ascending pain signals is another question. As shown in Fig. 1, PGH_2_ is subsequently metabolized into PGE_2_, PGD_2_, PGI_2_, or TXA_2_ by means of specific synthases. Accordingly, we here overviewed the actions of those four substances and then those of further metabolized PGs. Because PGD_2_ has often been comparably studied alongside PGE_2_, we present information about both of them here.

PGE_2_ is synthesized by the conversion of PGH_2_ through the action of one of three PGE_2_ synthases (PGESs), cytosolic PGES, membrane-bound microsomal PGES-1 (mPGES-1), and mPGES-2. Among the PGESs, one study showed that mPGES-1 was mainly coupled to COX-2-mediated PGE_2_ biosynthesis [46]. PGD synthase catalyzes another type of isomerization of PGH_2_ and two subtypes of PGD synthases have been identified [47]. The lipocalin-type PGD synthase (L-PGDS, also known as prostaglandin-H2 D-isomerase {PTGDS} or glutathione-independent prostaglandin D synthase) is an N-glycosylated enzyme that does not necessarily require free sulfhydryl cofactors such as glutathione (GSH) to execute its catalytic function and has been principally detected in the brain, testis, and heart [47, 48]. The other subtype of PGD synthase is hematopoietic PGD synthase (H-PGDS), which is a cytosolic GSH-dependent enzyme and is associated with the activity of GSH S-transferase. H-PGDS is expressed in immune cells, including mast cells and dendritic cells [47, 48]. According to the studies below, PG generation by mPGES-1 and L-PGDS in sensory neurons appears to contribute to inflammatory pain development.

### Formation of PGE_2_ and PGD_2_ in the DRG and spinal cord

#### PG production from infiltrated immune cells

When tissues are injured, prostaglandins produced by invading neutrophil and macrophage promote neuronal pain signals (see review: [49–51]). A recent work with transgenic mice whose the upstream transcription processes for PG-producing enzymes were genetically modified confirmed such an immune cell contribution in inflammatory pain models [52]. Moreover, both the neuronal and non-neuronal components of the peripheral somatosensory system have been shown to produce PGs.

#### PG production from neurons

During the early stage of research, chicken DRG were frequently used to gather information about PGs in the somatosensory system. Vesin and colleagues identified the PGs present in DRG homogenates from one-week-old chickens using a radio-labeled technique [53]. Two major PGs that were converted from the supplied [^14^C] arachidonic acid were [^14^C]PGE_2_ and [^14^C]PGD_2_, which indicated the presence of the enzymatic mechanism described above [53]. According to the same study, PGD_2_ was preferentially produced in primary somatosensory neurons, whereas PGE_2_ was mainly generated in fibroblast-enriched locations (i.e., meninges and DRG capsules) [54]. The location of PGD synthase in the DRG population was then investigated using immunohistochemistry [55, 56]. The immunoreactivity of L-PGDS was detected in 40% of small-diameter neurons, but in only 2% of large-diameter neurons, in DRG from 12-day-old chickens [55, 56]. The immunoreactivity of H-PGDS was detected in the satellite cells and Schwann cells that surround chick DRG neurons [55]. Therefore, the two isozymes of PGD synthase are involved in PGD_2_ formation in DRG and, in particular, GSH-independent L-PGDS is selectively expressed in the nociceptor subpopulation.

Soon after these initial findings from chicken nerves were reported, investigations using rodents began. In cultured embryonic rat sensory neurons, TNFα-induced capsaicin hypersensitivity decreased under indomethacin treatment, indicating that the cultured neurons could intrinsically produce prostaglandins [37]. Radioimmunoassays defined the presence of PGE_2_ and PGD_2_ in the lumbar spinal cords of rats [14]. RT-PCR and in situ hybridization assays conducted by Schuligoi et al. confirmed the expression of mPGES-1 and L-PGDS in the rat DRG and spinal cords, both of which were up-regulated after four hours of exposure to the endotoxin LPS [57]. An in vitro superfusion chamber study from the same group demonstrated that increases in PGE_2_ and PGD_2_ secretions could be observed in spinal cord slices from mice with systemic exposure to endotoxin for 24 h, and that those secretions were prevented by the addition of lumiracoxib (100 nM), a selective COX-2 inhibitor [13]. Grill et al. further demonstrated that the inflammation-induced promotion of PGD_2_ production depended on the action of COX-2, but not COX-1 [18]. In the spinal cords of those inflamed mice, the expression of mPGES-1 and L-PGDS was up-regulated probably by non-neuronal components [13, 18]. Those results indicate that the production of both PGE_2_ and PGD_2_ can be augmented via up-regulation of essential biosynthetic enzymes, such as the COXs, mPGES-1, and L-PGDS, in the DRG and spinal cord during inflammation. The expression of mPGES-1 in DRG neurons was later confirmed by immunohistochemistry [58], and that of L-PDGS has been confirmed in microarray and Western blot analyses [59].

Increased PGE_2_ levels were detected in injured peripheral nerves and their ipsilateral lumbar DRG 10 days after sciatic nerve injury, and they were suppressed by treatment with the non-selective COX inhibitor ibuprofen (40 mg/kg) [60]. Unlike the observations from chicken DRG, where PGE_2_ was hardly found in the neuronal components, the pro-inflammatory cytokine IL-1β facilitated PGE_2_ production in cultured rat DRG neurons through the extracellular signal-regulated kinase (ERK)/p38 mitogen-activated protein kinase (MAPK) pathway, presumably via elevated COX-2 expression [20]. In the aged rats with pSNL neuropathy mentioned above [45], chronic neuropathic pain was associated with heightened PGE_2_ levels in the injured nerve, caused by persistent COX-2 expression [45].

PGE_2_ was also found to be produced and secreted from the satellite glial cells surrounding the DRG neurons in response to fractalkine (also known as chemokine {C-X3-C motif} ligand 1 or CX3CL1), a chemokine released from DRG neurons when they are in an inflamed state [61]. Such evidence of the presence of PGE_2_ and PGD_2_ in the neuronal and glial components of the peripheral nociceptive pathway indicates that these PGs could considerably alter cellular functions. Receptor activation by secreted PGs may trigger those alterations.

#### PG production from non-neuronal cells

The data from a recent single-cell RNA sequencing study showed significant expression of the genes encoding PG-producing enzymes in not only neuronal, but also various non-neuronal components of a rodent brain, implying that secreted non-neuronal PGs might actively regulate neuronal functions [62]. In fact, multiple studies have confirmed that various non-neuronal cell types join in with PG formation in the peripheral somatosensory system (see review: [63]). For example, spinal astrocytes and microglia [64–66], Schwann cells of the sciatic nerve [67], and satellite glial cells surrounding trigeminal neurons [68] have been shown to produce PGE_2_ in the presence of tissue injury or inflammation. Taken together, PGs produced from neuronal, glial, and invaded immune cells locally play an important role in regulating the sensory neuronal function regarding pain signaling possibly in paracrine and autocrine manners.

### Genetic deletions of PGE_2_ and PGD_2_ synthases

mPGES-1 and L-PGDS, known as greater contributors to nociceptive processing among the PGE_2_ and PGD_2_-producing enzymes, were genetically deleted and tested in several pain behavioral studies. Nociceptive writhing was commonly reduced in three acetic acid-induced writhing models using mPGES-1-deficient mice [69–71]. Kamei and colleagues further observed reduced writhing responses in an LPS-primed condition [70]. The reduction in acetic acid-induced writhing responses seen in *Ptges* (which encodes mPGES-1 protein)-null mice was comparable to the effect seen with piroxicam when wild-type mice were treated [69]. Trebino et al. demonstrated that knockout mice displayed no significant difference in withdrawal latencies in hot plate assays compared with wild-types in the same study. In a different study, *Ptges*-null mice exhibited decreased neuropathic pain, such as mechanical allodynia and thermal hyperalgesia, under L5 nerve transaction [72]. This result is interesting because reducing the PGE_2_ level is currently not considered to be a best analgesic strategy for relieving neuropathic pain. Controversial results have also been produced in other pain behavioral models. *Ptges* knockout mice failed to show a difference in zymosan-evoked mechanical hyperalgesia and formalin-induced phase 1 and 2 nociceptive responses [71]. The shunting of the substrate to other PG synthases possibly explains that unexpected result because some other PGs also play pro-nociceptive roles, as explained below [73]. An interesting result was produced in one *Ptgds* (which encodes L-PGDS protein) knockout study in which intrathecally injected PGE_2_ caused heat hyperalgesia, but not mechanical allodynia, in *Ptgds*-deficient mice, suggesting that PGD_2_ generation at least partly contributes to the pro-nociceptive action of PGE_2_ [74].

### PGE_2_-activated EPs (PGE_2_ receptors)

#### EP expressions in the peripheral somatosensory system

**Fig. 2:**
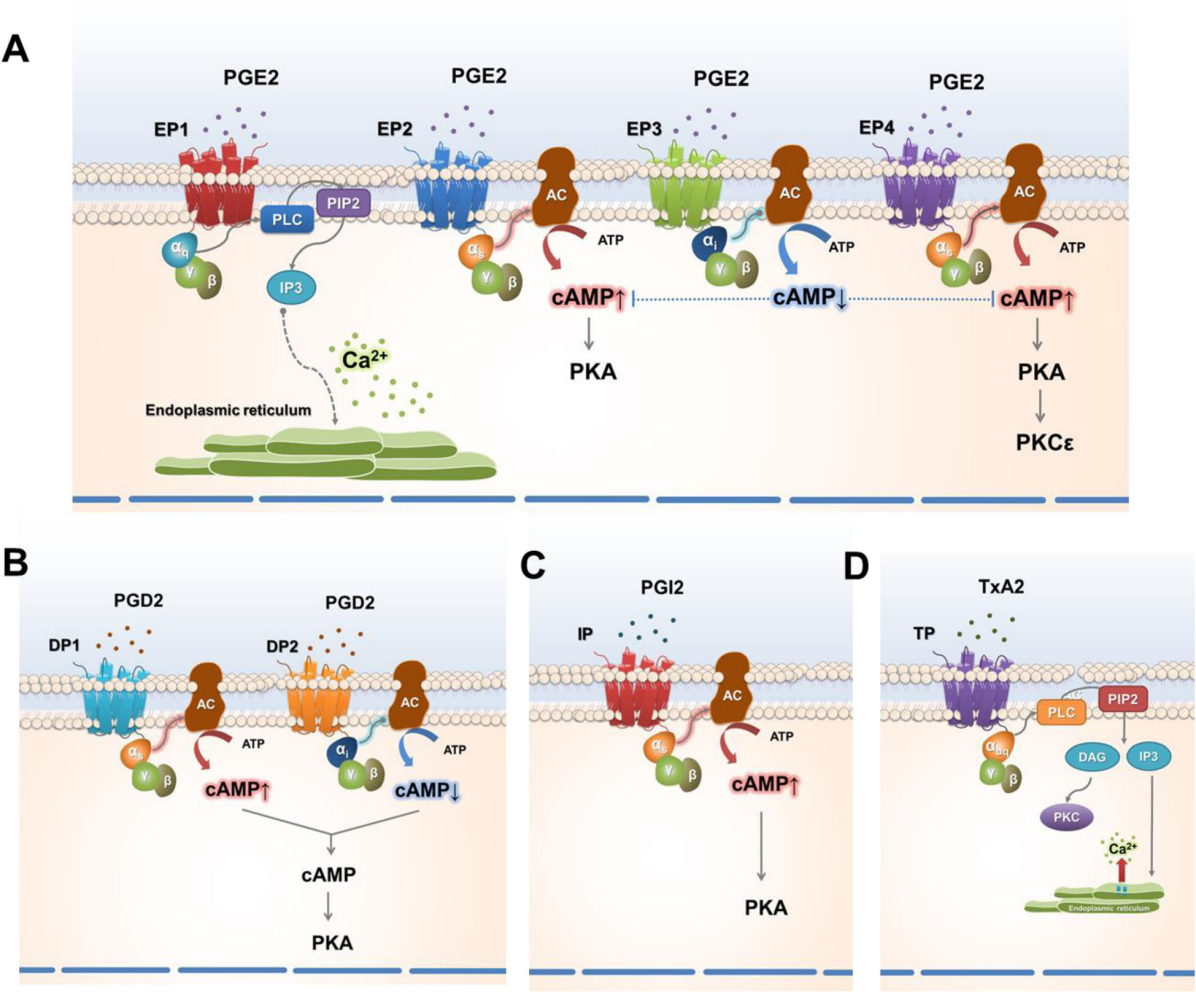
Signaling pathways initiated by prostanoids released from peripheral inflammation. **a** PGE_2_-induced activation of EP receptors in somatosensory neurons. In somatosensory neurons, the EP1 receptor is associated with the G protein, Gαq and its activation triggers the release of intracellular Ca^2+^ from the endoplasmic reticulum through inositol 1,4,5-trisphosphate (IP3) production. EP2 and EP4 receptors are coupled with Gαs, which stimulates cyclic adenosine monophosphate (cAMP) production by activating adenylyl cyclase (AC), whereas EP3 receptor-coupled Gαi activation inhibits cAMP production by inhibiting AC. cAMP in turn activates protein kinase A (PKA), causing phosphorylation of various signaling proteins including epsilon type protein kinase C (PKCε). **b-d** Signaling pathways initiated by the activation of DP, IP, and TP receptors in somatosensory neurons. The DP1-linked Gαs stimulates intracellular cAMP production, whereas DP2-associated Gαi inhibits cAMP production. The counter-action of DP1 and DP2 (**b**) receptors regulates cAMP accumulation in the cytosolic compartment. The activation of IP (**c**) or TP receptors (**d**) recruits Gαs protein, activates AC, and consequently raises the intracellular cAMP level

Secreted PGE_2_ interacts with its cognate G-protein coupled receptors, namely EP receptors located on the plasma membranes of neurons. The EP family is composed of four subtypes (EP1-EP4), each of which activates distinctive signaling pathways (reviewed in [75]). As shown in Fig. 2a, the EP1 receptor is associated with the Gαq G protein, and EP1 activation triggers the cytosolic release of Ca^2+^ from the endoplasmic reticulum through phospholipase C (PLC)-mediated inositol 1,4,5-trisphosphate (IP3) production [4, 76]. EP2 and EP4 receptors are coupled with Gαs which amplifies cAMP production by activating adenylyl cyclase. Reversely, EP3 receptor-coupled Gαi activation lowers the cAMP level by inhibiting adenylyl cyclase [4, 76]. In mouse DRG, in situ hybridization tracking EP expression offered the first evidence of mRNA transcripts of *Ptger1*, *Ptger3,* and *Ptger4* among the EP-encoding genes [77]. Later, *Ptger1*, *Ptger2*, *Ptger3C*, which is a *Ptger3* splicing variant, and *Ptger4* were detected in an RT-PCR analysis of rat DRG neurons [78]. More recently, *Ptger3A*, *B*, and *C* expressions were further confirmed in rat DRG [79]. In trigeminal neurons, EP2 and EP3 have been shown to be abundant in nociceptors [80]. The expression of EP2 in rat DRG was confirmed by a separate research group [81]. Although somatosensory EP expression itself has been shown repeatedly, its alteration in a pathological state remains unclear. Immunoreactivity to EP1 was temporarily increased and then returned to a normal level when the human brachial plexus nerve was injured [82]. In a rat model of cervical facet joint injury, which readily causes mechanical allodynia, EP2 expression in DRG neurons was elevated [81]. On the other hand, in vitro treatments of mouse DRG neurons with the pro-inflammatory cytokines, TNFα and IL-1β enhanced PGE_2_ production through the elevation of COX-2 expression, but there was no significant alteration in the mRNA levels of *Ptger1*- *Ptger4* [31]. A recent study showed that *Ptger2* but not *Ptger4* expression in DRG escalates in a murine model with endometriosis lesions [83].

#### Pharmacological evidence for EP expression

Pharmacological evidence of somatosensory EP expression has also been accumulating. A surgical incision in the middle of the L4-L6 spine lowered the mechanical threshold and was associated with heightened PGE_2_ levels in DRG neurons one to two weeks after surgery [84]. The sensitized mechanical response was reversed by oral administration of the EP1 antagonist, ONO-8713, for five days [84]. Direct injection of an EP1/EP2 (AH6809) or EP4 (AH2384810) antagonist into the L5 DRG significantly alleviated IL-1β-induced hyperalgesia in the hind paws of rats [21]. The EP3 agonist ONO-AE-248 mimicked PGE_2_-induced attenuation of the activity of voltage-gated Ca^2+^ channels in acutely isolated mouse trigeminal neurons [85]. The same EP3 agonist suppressed the PGE_2_-facilitated activity of a tetrodotoxin (TTX)-resistant voltage-gated Na^+^ channel (TTX-R) in cultured DRG neurons without altering the basal channel activity [79, 86]. Interestingly, a recent study demonstrated that one outcome from EP3 actions on DRG neurons could be pro-nociceptive because sulprostone, an EP agonist with higher efficacy on EP1 and EP3 than EP3 and EP4, induced the secretion of C–C motif-chemokine ligand 2 (CCL2) in DRG cultures from wild-type mice, whereas the effect was significantly milder in *Ptger3* (which encodes EP3 protein)-deficient mice. The data indicates that EP3-induced CCL2 secretion from DRG neurons could promote pain by activating C–C motif chemokine receptor 2 (CCR2), which is expressed in spinal neurons and microglia [87].

The presence of EPs in the spinal cord has been implied mostly by pharmacological evidence. The spinal application of an EP1 (ONO-DI-004), EP2 (butaprost), or EP4 (ONO-AE1–329) agonist caused dorsal horn hyperexcitability in an in vivo electrophysiology test when the relevant knee joint and ankle were mechanically stimulated under normal conditions [86]. For an inflamed knee joint, only spinal application of the EP1 agonist (ONO-DI-004) further facilitated the hyperexcitability of the spinal dorsal horn neurons, whereas the application of the EP3 (ONO-AE-248) and EP4 agonists (ONO-AE1–329) did not [79, 86]. The same application of the EP3 agonist (ONO-AE-248) reversed the hyperexcitability caused by knee joint inflammation, which could be underpinned by Gαi-coupled signaling [86]. Similarly, intrathecal administration of an EP1 antagonist (ONO-8711) in rats blunted PGE_2_-induced mechanical hyperalgesia in a dose- and time-dependent manner [88]. It is intriguing that this study demonstrated *Ptger1* expression only in the DRG and failed to show it in the spinal cord, using in situ hybridization, suggesting that EP1 might facilitate presynaptic signals in the central termini of DRG neurons. An intracellular calcium imaging analysis of spinal cord slices from the same study showed that ONO-8711 largely inhibited the PGE_2_-induced Ca^2+^ influx in laminae II–VI in the dorsal horn, which could result from decreased presynaptic functions [88]. Collectively, these data demonstrate that EPs are expressed in peripheral somatosensory components, and EP1, EP2, and EP4 in particular appear to play primarily pro-nociceptive roles.

#### Kinase-mediated EP signal transduction

Studies investigating the functional roles of EPs have also expanded knowledge of the downstream information regarding the actions of protein kinases. Many types of DRG neuronal ion channels that confer electrical excitability experience phosphorylation of their intracellular amino acids, which frequently leads to enhanced channel activity. EP-induced signaling mediates phosphorylation by PKA and PKC (Fig. 2). Detailed mechanisms are mentioned below for the facilitated activities of TTX-R, voltage-gated Ca^2+^ channels, purinoceptors, and TRP channels. Interestingly, there appears to be modulation between PKA and PKC actions in EP signaling. Gold and colleagues suggested that PKC activity appears to be necessary for PKA-mediated positive modulation of TTX-R activity [89]. A differential result has also been reported. In the L4-L5 DRG, an in vivo intraplantar injection of PGE_2_ (100 ng per paw) robustly elevated not only PKA activity 30 min later, but also PKCε activity three hours after injection [90]. This in vivo study further showed that the effect on PKCε disappeared when PKA was pharmacologically inhibited, suggesting that PKA is able to regulate PKCε activity [90].

Janus kinase 2 (JAK2) is a cytoplasmic tyrosine kinase that is activated through pro-inflammatory cytokine signaling [91]. A JAK2-induced transcriptional cascade has been shown to be involved in inflammatory and neuropathic pain [92, 93]. Interestingly, a recent report has demonstrated that PKCε is a merger point for the actions of PGE_2_ and JAK2 [94]. Intrathecal injection of the JAK2-selective inhibitor AG490 blocked the membrane translocation of PKCε in the L5 DRG in a carrageenan-inflamed hind paw model [94]. The same administration also blunted the hyperalgesia induced by an intraplantar injection of PGE_2_ or carrageenan [94]. Furthermore, they showed that an in vitro pre-incubation of DRG neurons with AG490 prevented the potentiation of a TRPV1-mediated Ca^2+^ influx by PGE_2_. Although the transcriptional link associated with JAK2-induced PKCε translocation remains elusive, that study again emphasizes the importance of PKCε as a signal transducer for the pro-nociceptive actions of PGE_2_ and suggests that JAK2 could intervene in that process.

Nitric oxide (NO) has been suggested as a way to mediate PKA signaling. The intradermal injection of PGE_2_ in the paws of rats caused mechanical hyperalgesia, which was inhibited by the NO synthase (NOS) inhibitor N^G^-monomethyl-L-arginine (L-NMMA) [95]. L-NMMA also inhibited mechanical hyperalgesia induced by the stimulation of PGE_2_ downstream steps such as injections of 8-bromo-cAMP (a stable membrane-permeable analog of cAMP) or forskolin (an adenylyl cyclase activator). However, it failed to alter hyperalgesia produced by the injection of the PKA catalytic subunit, indicating that NO could play a role in the interaction between adenylyl cyclase and PKA [95].

EP4 also appears to regulate cytokine expression. Normally, interleukin-6 (IL-6) is detected in a very small fraction of C-nociceptor neurons. pSNL neuropathy causes expanded IL-6 expression in a wider subset of DRG populations, which has been shown to be mediated through EP4 activation [96]. The incubation of cultured DRG neurons with PGE_2_ resulted in elevated IL-6 expression, which depended on EP4 activation and kinase activation [96]. Tse and collogues revealed an interactive event between the activation of EP4 and toll-like receptor 4 (TLR4) in their series of studies although much remains be explored regarding the kinase action: LPS-induced TLR4 activation regulated the production of PGE_2_ in the sensory neurons and glial cells of rat DRG [97, 98]. Also, DRG neuron-derived PGE_2_ modulated TLR4 activity dependent on the activation of EP4 in DRG neurons and glial cells in an autocrine and paracrine manner [99].

#### Differential pro-nociceptive roles of EP2 and EP4

The role of EP2 in PGE_2_-induced pro-nociception has mainly been studied in the spinal cord. Treatment with the EP2 agonist butaprost induced an inward current in the deep dorsal horn neurons of rats in a way similar to that seen with PGE_2_ exposure [100]. The Zeilhofer group reported a different aspect of EP2 activation that led to a Gs-dependent reduction in the glycinergic inhibitory transmission in rat superficial dorsal horn neurons [101]. Two years later, a study using zymosan and CFA-induced mouse inflammation models confirmed that such EP2-mediated interruption of the spinal glycinergic transmission contributed to inflammatory pain [102]. This spinal EP2-induced pro-nociceptive paradigm was again confirmed with Ptger2 (which encodes EP2 protein) knockouts in the inflammatory pain model [103], but not in the formalin-induced pain model or a neuropathic pain model [104]. Two recent studies reported interesting findings: spike timing-dependent long-term potentiation occurred in the lamina I spinal projection neurons of female mice in an EP2-dependent manner in a study using the EP2 selective antagonist PF 04418948 and the agonist butaprost, which awaits the quantification of in vivo pain contribution in comparison to the above findings from the effects on the superficial inhibitory and deep excitatory circuits [105]. Elevated *Ptger2* expression in the DRG was recently shown in a murine endometriosis model. In the same study, EP2 antagonism, targeting receptors that probably include the ones expressed in DRG, was more effective in reducing both primary and secondary hyperalgesia than EP4 antagonism [83].

In DRG neurons, numerous studies focused first on PGE_2_-mediated EP4 activation. It was determined that PGE_2_ and PGE_1_ (which is not derived from arachidonic acid but from dihomo-γ-linolenic acid) induced cAMP accumulation by stimulating adenylyl cyclase in rat DRG neurons [106, 107]. Only the EP4 agonist (ONO-AE1–329) caused intracellular cAMP accumulation in adult rat DRG neurons, while EP1 (ONO-DI-004), EP2 (ONO-AE1–259-01), and EP3 (ONO-AE1–329) agonists did not [108]. Interestingly, only EP4 expression, not EP1–3 expression, was elevated in L5 DRG neurons following CFA-induced unilateral hind paw inflammation in a different study [109]. Lin et al. also demonstrated that silencing EP4 action with an intrathecally administered EP4 antagonist (AH23848) or with its knockdown using short hairpin RNA (shRNA) rescued thermal and mechanical hypersensitivity without changing basal pain behavioral sensitivity in this rodent peripheral inflammation model [109]. AH23848 also suppressed PGE_2_-induced sensitization of the nociceptive ion channel TRPV1 to its agonist capsaicin in DRG neurons [109]. St-Jacques and Ma have shown that PGE_2_-mediated EP4 activation led to increased translocation of the EP4 protein to the plasma membrane of DRG neurons. They also demonstrated that EP4 recycling in DRG neurons is facilitated during inflammation, which enhances PGE_2_ sensitivity [110, 111]. Another study by that group showed PGE_2_ can facilitate anterograde axonal trafficking of the EP4 protein, resulting in increased EP4 availability at the axonal terminal of nociceptor neurons [112]. The expression of EP4 was also found in glial cells isolated from the DRG, and its functionality was confirmed by detecting specific agonist-induced cAMP production that was preventable by antagonist application [113].

### PGD_2_-activated DPs (PGD_2_ receptors)

The presence of DP receptors in sensory neurons was first predicted by pharmacological studies that showed mild cAMP accumulation in DRG neurons after PGD_2_ exposure [106, 107]. PGD_2_-evoked CGRP release from trigeminal neurons was prevented by pre-incubation with the DP1 antagonist BWA868C [114]. PGD_2_ binds to and activates DP receptors, which have two subtypes: DP1 and DP2 (Fig. 2b). The DP1-linked Gαs stimulates adenylyl cyclase activity in the same manner as the EP2 and EP4 receptors, whereas DP2-associated Gαi inhibits the same enzyme as the EP3 receptor (Fig. 2). Therefore, cAMP accumulation and CGRP secretion implies that DP1 action is predominant in sensory neurons. A decade after that research, DP1 and DP2 proteins were shown to be broadly detected in small to large-sized neurons of the lumbar DRG [115]. Ebersberger and colleagues also pharmacologically confirmed that a selective DP1-specific agonist (BW245C) augmented the excitability of DRG neurons by facilitating both the conductance and voltage-dependence of TTX-R, which is presumably encoded by the Nav1.8 or Nav1.9 genes. On the other hand, treatment with the DP2 agonist 15(R)-PGD_2_ occasioned no response. Interestingly, the DP2 agonist interfered with the Na^+^ conductance mediated by DP1 activation, suggesting that DP2 functions as a negative regulator of DP1 activation [115]. Because the net effect of PGD_2_ treatment is the enhancement of the Na^+^ current, PGD_2_ appears to be pro-nociceptive, and DP1 may predominantly mediate that effect in these afferent nociceptors. A quantitative real-time RT-PCR analysis confirmed the predominance of *Ptgdr1* (encoding DP1) expression over *Ptgdr2* expression in both DRG and trigeminal neurons [116]. Nociceptor-specific *Ptgdr1* expression has been replicated using different methods, such as a transcriptomic analysis using DRG neurons and an in situ hybridization assay using trigeminal neurons [117, 118]. Unlike the data for DP receptors in DRG, results concerning spinal DPs have been relatively confusing. *Ptgdr1* and *Ptgdr2* expression was detected in murine spinal dorsal horn neurons [18]. Interestingly, the mRNA levels of both *Ptgdr1* and *Ptgdr2* increased under systemic inflammation induced by the intraperitoneal injection of an endotoxin (1 mg/kg), but their protein levels did not [18]. Telleria-Diaz et al. conducted in vivo spinal recordings and showed that DP1 activation prevented electrical discharges, and DP1 inhibition promoted them when inflamed knee joints were mechanically stimulated [119]. It is possible that spinal interneuron subsets serve differential roles using the DP1 receptor.

### Pro-nociceptive effectors of PGE_2_ signaling

**Fig. 3:**
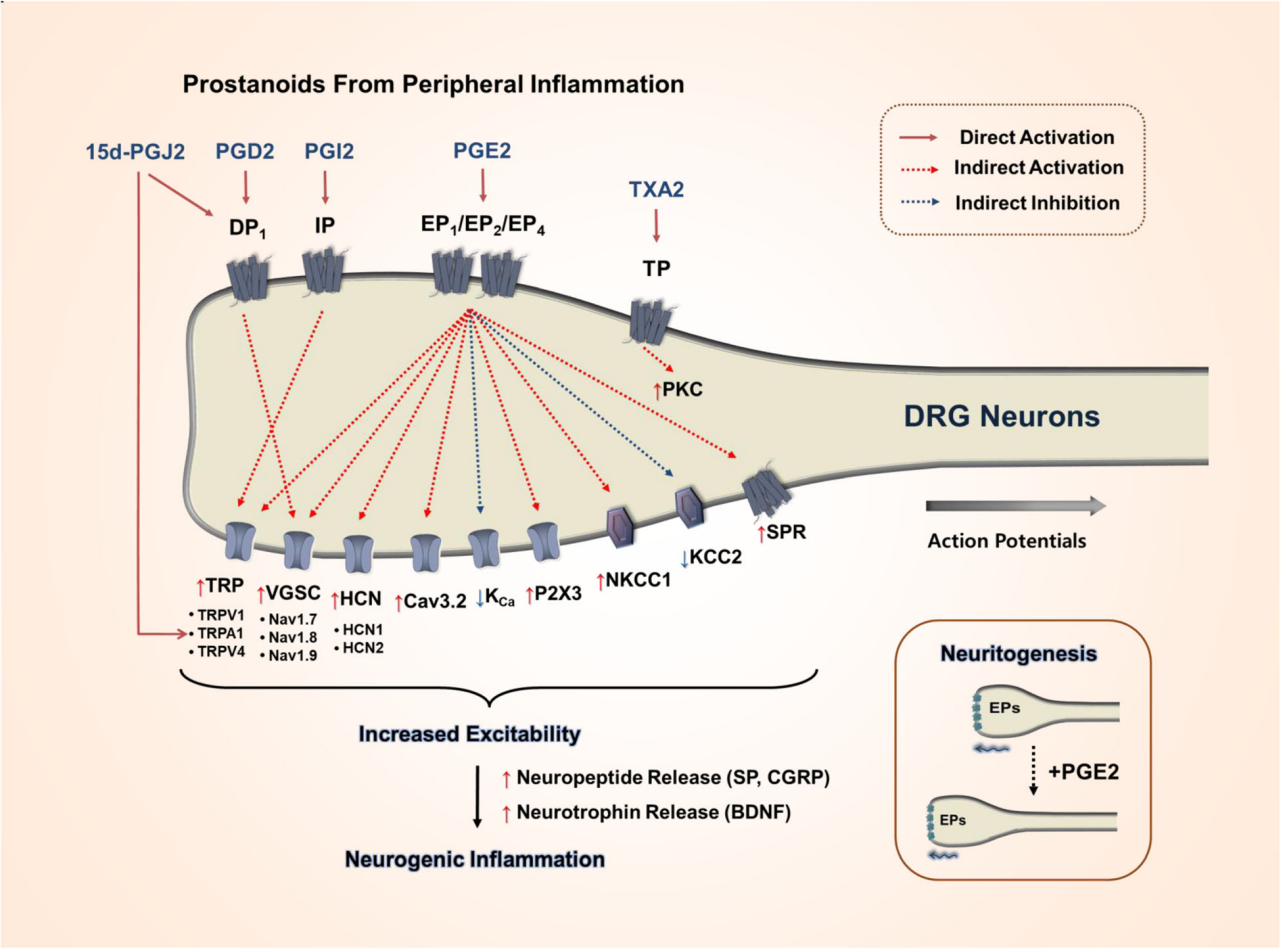
Pro-nociceptive effector molecules that contribute to pain exacerbation by PGs in somatosensory neurons. The functions of diverse ion channels, transporters, and metabotropic receptors are altered by the signal transductions described in Fig. 2, eventually promoting the electrical excitability, neurogenic inflammation, and neuritogenesis of somatosensory neurons

As mentioned above, EP1–2, EP4, and DP1-mediated signaling is predominant in directing the action of PGE_2_ and D_2_ to pro-nociceptive outcomes in the peripheral somatosensory system. Those outcomes are finally achieved by increasing the excitability and secretability of nociceptors. The molecular effectors of this increased excitability and secretability have been studied mainly with regard to the actions of PGE_2_ and are categorized below (Fig. 3).

#### The inhibition of slow spike after-hyperpolarization (AHP_Slow_) by PGE_2_

In 1976, Coleridge and colleagues first reported the promotive effects of PGE_2_ on C-fiber impulses in the lungs of anesthetized dogs [120]. Since then, many studies have investigated the effects of prostaglandins on visceral afferent neurons. The application of PGE_1_, PGE_2_, and PGD_2_ (but not PGF_2α_) attenuated Ca^2+^-dependent AHP_Slow_, leading to increases in the excitability of the C-fibers in the leporine nodose ganglion [121, 122]. The inhibitory action of PGs on AHP_Slow_, which is known to use the intracellular Ca^2+^-activated K^+^ channel (K_Ca_), appeared to be independent of their effect on extracellular Ca^2+^ influx, suggesting that other mechanisms, such as an intracellular signaling, may be important [123]. As a final outcome, the inhibition of AHP_Slow_ by PGE_2_ and PGD_2_ caused membrane excitability to increase due to augmented membrane resistance and depolarization to an extent [122]. That contribution of AHP_Slow_ was partly replicated in cultured rat DRG neurons [124]. Essentially, only a subpopulation of nociceptors exhibited AHP_Slow_. PGE_2_ suppressed AHP_Slow_ in those nociceptors, which increased the frequency of action potentials [124]. It remains elusive which subtypes of K_Ca_ channels are inhibited by PGE_2_ action. Although AHP_Slow_ is known to be uninvolved in setting the threshold for action potential generation, PGE_2_ exposure also reduced that threshold [124]. Therefore, those authors suggested that PGE_2_ may also modulate other electro-excitatory molecules in addition to AHP_Slow_, which is currently considered TTX-R [124].

#### The augmentation of TTX-R Na^+^ currents by PGE_2_

Voltage-gated Na^+^ channels (VGSCs) play an essential part in the initiation and propagation of action potentials in neurons. Sensitivity to TTX in its blockade of VGSCs has long been a pharmacological standard for subcategorizing VGSCs. In small-diameter DRG nociceptors, PGE_2_ has been shown to increase the magnitude of TTX-R Na^+^ currents (TTX-R I_Na_) and causes a hyperpolarizing shift in its steady-state inactivation curve, which was shown to depend on the cAMP-PKA pathway [125]. The cAMP-dependent phosphorylation of Nav1.8, most prevalently responsible for TTX-R I_Na_, caused hyper-excitability in membrane potential recordings of COS-7 cells heterologously overexpressing Nav1.8 [126]. Gold and colleagues found that not only PKA but also PKC contribute to the positive modulation of TTX-R activity in rat DRG neurons [89]. Interestingly, they suggested that PKC activity might be required for PKA-mediated modulation of TTX-R I_Na_ [89]. PGE_2_ application has also been shown to promote TTX-R I_Na_ in endogenous Nav1.8 and Nav.1.9 channels in small-diameter DRG neurons [127, 128]. Moreover, Rush and colleagues have shown that PGE_2_ enhances Nav1.9-specific Na^+^ currents in Nav1.8-deficient DRG neurons [129]. Jang and colleagues recently showed that PGE_2_ potentiates GABA_A_–mediated Ca^2+^ transients and membrane depolarization in nociceptive DRG neurons via EP4 activation [38]. This potentiation does not seem to be caused by directly altering GABA_A_ activity or intracellular Cl^−^ homeostasis, but by increasing Nav1.8 activity [38]. TTX-S in Aδ nociceptors also appears to be sensitized in an adenylyl cyclase-PKA dependent fashion, which awaits replication [130, 131].

In vivo models have also corroborated the effect of PGE_2_ on TTX-R I_Na_. The intrathecal injection of an antisense ODN for Nav1.8, resulting in its knockdown, attenuated PGE_2_-induced hyperalgesia in rats [132]. The same study confirmed that in vitro treatment of cultured DRG neurons with an ODN for Nav1.8 selectively lowered TTX-R I_Na_ density compared with untreated neurons [132]. Longitudinal incisions on rat hind paws caused a decrease in mechanical withdrawal thresholds and an increase in TTX-R I_Na_ in DRG neurons [27]. One day and two days after the incision was made, the concentrations of PGE_2_ and CGRP were both significantly elevated in the paw tissue and DRG neurons. When the rats were orally treated with celecoxib (30 mg/kg) one hour before and 12 h after incisional surgery, mechanical pain behaviors, TTX-R I_Na_, and the concentrations of PGE_2_ and CGRP were commonly down-regulated [27]. Another in vivo model proposed a new molecular mechanism for this augmentation of TTX-R currents. The subcutaneous injection of CFA aggravated mechanical and thermal pain behaviors and also led to the increased expression of Nav1.7 and Nav1.8 in DRG neurons [32]. Those authors suggested that PGE_2_ contributes to this elevated expression of the TTX-Rs by showing that oral administration of COX inhibitors, either ibuprofen (200 mg/kg) or NS-393 (10 mg/kg), blunted both the elevation of TTX-R expression and the heightened pain behaviors [32]. Direct PGE_2_ exposure replicated the increased transcription and translation of Nav1.7 in an explant culture of trigeminal ganglia, which appeared to be mediated by EP2 activation [133]. Interestingly, increased protein translocation also seems to be involved. It has been demonstrated that PGE_2_-activated PKA directly phosphorylates the RRR motif of the first intracellular loop of the Nav1.8 channel protein, facilitating the membrane localization of Nav1.8 [134]. Deletion of the Nav1.8 gene, however, failed to generate firm evidence. PGE_2_-induced hypersensitivity and neuropathic pain behaviors in mice were unaltered by a Nav1.8-null mutation [135]. These results suggest that the compound actions of other effectors for neuronal excitability including those of multiple types of TTX-R may be required for the pro-nociceptive function of PGE_2_.

Despite the small number of studies conducted, PGD_2_ has also been shown to increase the conductance and maximal current amplitudes of TTX-R I_Na_ in rat adult DRG neurons by means of Nav1.8 or Nav1.9 activation [115]. The specific activation of Gαs-coupled DP1 receptors appears to be required for this facilitation, which was neutralized by the activation of the Gαi-coupled DP2 receptor [115]. Thus, PGD_2_ may regulate TTX-R I_Na_ through a balance of DP1 and DP2 receptor activation.

#### The effect of PGE_2_ on Cav3.2 voltage-gated Ca^2+^ channels

Sekiguchi and colleagues have confirmed that the EP4-cAMP- PKA axis is responsible for PGE_2_-induced mechanical hyperalgesia [136]. They suggested that one of the final molecular effectors for this pathological pain is the T-type voltage-gated Ca^2+^ channel (Cav3.2) of nociceptors [136]. Voltage-dependent responses of Cav3.2 were sensitized by PKA-catalyzed phosphorylation. Furthermore, A-kinase anchoring protein 150 (AKAP150), bound directly to Cav3.2, has been shown to facilitate the action of PKA [136].

#### The effect of PGE_2_ on purinergic P2X purinoceptors

Adenosine triphosphate (ATP) in the peripheral somatosensory system has been recognized as an important inflammatory mediator that evokes nociception [137]. Cation influx through the ionotropic ATP receptor P2X, when activated by binding to extracellularly secreted ATP, causes neuronal depolarization, which leads to the generation and exacerbation of pain signaling. Interestingly, this ATP-mediated hyperalgesia was potentiated by co-injection with PGE_2_ [138]. Later studies have explored the molecular mechanism of this pathway. Wang et al. showed that PGE_2_-mediated cAMP production contributes to P2X activation in DRG neurons [139]. Interestingly, the crucial receptor subtype associated with PGE_2_ action for P2X activation was found to be EP3, which is known to down-regulate cAMP in general [139]. Using their P2X3 potentiation model, Wang and colleagues also suggested that a mechanism for the signaling switch from PKA to PKC, which has been demonstrated in many other PGE_2_ studies, is mediated by the cAMP-responsive guanine nucleotide exchange factor 1 (Epac) protein [140]. Recently, the same group has further shown that Epac-mediated PKC signaling facilitates the membrane expression of P2X3 by increasing F-actin levels in DRG neurons, contributing to PGE_2_-sensitized P2X3 currents [141]. Pharmacological evidence for P2X3 involvement has also been provided. P2X3 expression was augmented in rat DRG neurons in a chronic constriction injury (CCI) neuropathic pain model and returned to a normal level after treatment with ibuprofen and celecoxib [28]. PGE_2_-induced hyperalgesia was relieved by treatment with a P2X3 inhibitor (A317491) or by P2X3 knockdown using an antisense ODN [142]. In the same study, PKCε was determined to be the most critical isozyme of PKC in P2X3-mediated hyperalgesia. Taken together, these findings indicate that PGE_2_ facilitates the action of P2X3 via the sequential activation of PKA and PKC signaling, which produces sensitized responsiveness to ATP, an important inflammatory mediator.

#### The effect of PGE_2_ on the TRPV1 channel

TRPV1 is considered to be the most important receptor-ion channel expressed in the Aδ- and C-fibers of DRG. TRPV1 activation depolarizes the nociceptor population via cation influx in direct response to diverse harmful stimuli such as noxious heat, lipid peroxides, and leukotrienes and pungent chemicals such as capsaicin and tarantula toxin, generating and exacerbating pain [143–145]. In addition, TRPV1 activation in dorsal horn neurons is commonly suggested to contribute to pain transmission [146, 147]. Furthermore, many inflammatory mechanisms have been shown to augment TRPV1 activity, which explains an important aspect of inflammatory pain. For example, BK, histamine, TNF, and nerve growth factor (NGF) activate and/or sensitize TRPV1 via their specific signal transductions that use PLC, phospholipase A2 (PLA2), phosphoinositide 3-kinase (PI3K), and MAPK [148]. In this context, there have been important findings in PG research exploring whether and how PG signals and TRPV1 are linked, particularly regarding the actions of PGE_2_ and PGI_2_.

Even before TRPV1 was cloned, the Levine group had shown that PGE_2_, as well as PGI_2_, potentiates capsaicin-induced currents in adult rat DRG neurons and also successfully replicated that potentiation effect using cAMP analogs [149]. Similar results were obtained in a study that better mimicked physiological PGE_2_ and cAMP concentrations and conducted single channel recordings [150]. Comprehensive investigations into TRPV1 signaling were completed after the TRPV1 gene and protein were identified. Using nociceptors cultured from wild-type animals and TRPV1 and EP transgenic knockouts, heterologous expression platforms transfected with either intact or phosphorylation-resistant TRPV1 clones, and specific pharmacological agents, the Tominaga group has thoroughly observed the signal transduction of TRPV1 [151]. As a result, they determined that the activation of EP1/EP4 and IP (prostaglandin I2 receptor) was critical for TRPV1 sensitization by PGE_2_ and PGI_2_, respectively, and that the specific PGs do not show strong cross-reactivity for their receptor activation [151]. In the same study, TRPV1 phosphorylation by PKCε and PKA were both important downstream effects, but the time-scales for the peak effects differed: PKCε-mediated potentiation occurred first, around one minute after PGE_2_ exposure; several minutes later, PKA action began. They confirmed that Gαq-coupled PKCε activation depends on preceding PLC activation whereas Gαs-coupled PKA activation depends on cAMP production. This time-differential activation was more prominent in IP activation by PGI_2_, because nanomolar concentrations of PGI_2_ induced only the PKA-dependent slow effect whereas higher concentrations produced both the slow effect and the PKC-mediated fast effect, confirming an earlier biochemical study that had used other tissues [152] (Namba et al., 1994). Such potentiation occurred not only in capsaicin responsiveness, but also in the heat responsiveness of TRPV1, which displayed a reduction in the temperature threshold of at least 10 degrees [151]. This appears to be a striking finding because the data indicate that prostaglandin-mediated inflammation can cause constant pain in response to normal body temperatures via TRPV1 potentiation.

More evidence for TRPV1 as an effector of prostaglandin signaling has been further reported. Two independent groups have demonstrated that AKAP150, which was also reported to be important for Cav3.2-mediated action, is essential for TRPV1 sensitization by PGE_2_ in DRG neurons and trigeminal neurons [153, 154]. AKAP150 was shown to physically bind to the TRPV1 protein. Once bound, PKA can anchor it in place, which could raise the efficiency of the PKA approach in targeting TRPV1 sequences for phosphorylation. Very recently, PGE_2_ has also been shown to elevate TRPV1 expression and further promote its translocation to the plasma membrane [155]. Therefore, PGE_2_ appears to facilitate the expression, trafficking, and activity of TRPV1, which promotes the nociceptive signaling of somatosensory neurons. Unlike the PGE_2_-TRPV1 relationship, few studies have examined the functional interaction between PGD_2_ and TRPV1 in the somatosensory system. The Hucho group recently emphasized the possible involvement of PGD_2_ by showing that the DP1 receptor is highly enriched in the TRPV1-positive subpopulation of nociceptors compared with other subsets [117]. Intriguingly, those authors also suggested that PGD_2_ secreted from large-diameter Aβ fibers, which displayed higher expression of L-PDGS than nociceptors in their transcriptomic analysis, may act on the DP1 of TRPV1-positive nociceptors in a paracrine manner.

#### The effects of PGE_2_ on other TRP channels

Other nociceptive TRP channels appear to experience similar sensitization upon PGE_2_ exposure. The activity of TRPV4, which is responsible for detecting noxiously mechanical stretches and hypoosmolality, was first shown to be augmented by PGE_2_ exposure in a series of studies conducted by the Levine group [156–160]. They used this facilitation paradigm to establish an in vivo TRPV4-mediated pain behavioral model, in which rodents primed acutely by intraplantar injection of PGE_2_ exhibited hypoosmolality-induced flinches that were not readily observed in unprimed animals probably due to the presence of TRPV4 in a very small subset of nociceptors [161]. The Hwang group has further shown the utility of that model in screening TRPV4 modulators [162–165].

The contribution of the ankyrin subtype 1 of TRP (TRPA1) to PGE_2_-induced hyperalgesia has been reported. TRPA1 is a nonselective cation channel expressed in a subset of C-fibers and is comparable to TRPV1 in its extensive coverage of its sensible stimuli which are all painful. For example, TRPA1 is activated by noxiously cold temperatures (< 17 °C), mechanical stretches, and endogenous and exogenous irritants (e.g. lipid peroxides, allyl isothiocyanate {AITC}, cinnamaldehyde, and acrolein) [166, 167]. Dall’Acqua et al. demonstrated that PGE_2_-mediated hyperalgesia is blunted by both pharmacological and genetic inhibition of TRPA1 [168]. Hyperalgesia induced by PKA and PKCε was also reduced by TRPA1 inhibition, suggesting that those enzymatic processes are involved in TRPA1’s contribution to pain signaling [168].

#### The regulation of cellular cl^−^ homeostasis by PGE_2_

The intracellular Cl^−^ level determines whether and to what extent the activation of Cl^−^ channels such as GABA_A_ receptors and anoctamins, hyperpolarize or depolarize the membrane potential [169]. When the intracellular Cl^−^ concentration is relatively high, the channel activation allows Cl^−^ ions to diffuse to the outside of the cell and drives depolarization of the cell membrane, which typically occurs in DRG neurons [169, 170]. An inflammatory soup containing micromolar PGE_2_ has been shown to cause even higher intracellular Cl^−^ concentrations in rat DRG neurons by inversely regulating the protein levels of the two essential Cl^−^ transporters that maintain Cl^−^ concentration homeostasis in the following manner: increasing protein levels of the Cl^−^ importer Na-K-Cl cotransporter 1 (NKCC1) and decreasing the levels of the Cl^−^ exporter K-Cl cotransporter 2 (KCC2) [171]. Those authors suggest that nociceptive signals caused by Cl^−^ currents could be augmented. However, a more recent study conducted by the Oh group demonstrated that *Slc12a2* (which encodes NKCC1 protein)-null mice show no difference in the PGE_2_-induced potentiation of GABA_A_-mediated nociception compared with wild type mice [38]. It remains controversial whether DRG express KCC2 and which KCC subtypes are predominantly expressed [172–177]. More thorough approaches, such as using specific PGs, precisely monitoring KCC protein levels, and screening the effective duration of their exposure for altering Cl^−^ homeostasis could be required in the future.

#### The effect of PGE_2_ on hyperpolarization-activated cyclic nucleotide gated channels (HCNs)

HCN ion channels are activated by hyperpolarized voltages or cAMP and lead to cation influx, depolarizing the membrane potential. Therefore, their activation increases neuronal excitability and, in particular, contributes to the elevation of action potential frequency [178]. Among the four isotypes, HCN1 and 2 appear to be highly expressed in DRG neurons [179–182]. Given their intrinsic sensitivity to cAMP, HCNs had been hypothesized to be activated by cAMP produced by PGE_2_ signaling, bypassing further signal transduction. Indeed, even before HCN gene identification, hyperpolarization-activated cation current (Ih) in cultured nodose neurons was shown to be greatly enhanced by PGE_2_ exposure [183, 184]. More than a decade later, the subtypes that are most reactive to PGE_2_ in DRG neurons were confirmed. An *Hcn1* knockout study conducted by the McNaughton group demonstrated that HCN1, which was mainly expressed in large-diameter neurons, was activated by PGE_2_-produced cAMP, and that this mechanism was at least partly responsible for cold allodynia caused by pSNL neuropathy [181]. Using Nav1.8-positive nociceptor-specific knockouts for *Hcn2* (instead of global *Hcn2* knockouts, because those were extremely unhealthy), the same group further showed that HCN2 is activated in the same manner and contributes to heat, but not mechanical, hyperalgesia in a PGE_2_ injection model, carrageenan-inflammation model, and CCI neuropathy model [185].

Large-diameter Aβ afferent neurons, which normally relay casual touch signals as mentioned above, can become hyper-excitable and take part in nociception in certain pathological conditions [186–188]. Such an event is one cause for mechanical allodynia, in which light touch stimuli are misinterpreted as painful. In this situation, PGE_2_ seems to play a role. A recent study found that COX-1-generated PGE_2_ sensitizes Aβ DRG neurons and eventually contributes to mechanical allodynia in a bee venom injection model with the ablation of TRPV1-positive neurons [39]. The sensitizing mechanism appears to occur through up-regulated expression of HCN1 and 2. Consequently, Ih was significantly increased in the large-diameter neurons, and as a result, those neurons changed their action potential firing pattern from phasic to sustained. How the elevated Aβ signals can be transmitted to the pain perception center remains as a current issue for further investigations [189].

#### Reciprocal effects of PGE_2_ and BK

BK promotes inflammation and inflammatory pain by increasing vasodilation, vascular permeability, mediator synthesis, and nociceptor excitability [190]. In fact, numerous studies have hypothesized that BK and PGE_2_ interact closely in processing inflammatory pain. As a result, it is currently known that BK facilitates the production and release of PGE_2_, and that BK-induced nociception is also synergized by the addition of PGE_2_. Through the signal transduction and effector mechanisms listed above, PGE_2_ facilitates nociceptor excitation rather than directly causing action potentials in those neurons. BK-induced excitation is affected in the same way [191]. Interestingly, Smith and colleagues have mechanistically shown that this process may involve the mobilization of Ca^2+^, which is a central second messenger for PGE_2_ signal transduction [192]. In a subpopulation of capsaicin-responsive small-diameter DRG neurons, PGE_2_ increased intracellular Ca^2+^ in a manner that depended on the presence of extracellular Ca^2+^ [192]. Through this mechanism, pre-incubation with PGE_2_ potentiated a BK-induced intracellular Ca^2+^ increase and BK-evoked SP release in small-diameter nociceptors, as well as an increase in the number of BK-responsive neurons [192]. This sensitizing effect was reproduced by the application of bucladesine, a membrane-permeable cAMP analog, and the effect was suppressed by H89, a PKA inhibitor [192]. It is possible that the activities of voltage-gated Ca^2+^ channels or Ca^2+^-permeable TRPV1, all of which are effectors for PGE_2_ signaling, are positively modulated by PKA action that thereby contributes to Ca^2+^ influx. Thus, the critical point for signal merge appears to be an increase in intracellular Ca^2+^, which is also essential to BK-induced signal transduction. In the same study, PGI_2_ treatment exerted a similar effect on the BK-induced Ca^2+^ increase, whereas PGF_2α_ treatment did not [192]. PGE_2_ elevated the magnitude of depolarization and increased the number of action potentials induced by BK [193]. Such PGE_2_-induced potentiation was independent of the concentration of NGF, which can also induce inflammatory pain and the hypersensitivity of somatosensory neurons [193, 194].

Several studies have emphasized that BK uses PGs for one of its final outcomes, pain induction. The COX signaling pathway appears to be required in developing BK-induced mechanical hypersensitivity [40, 195]. BK has been shown to lead to COX induction and this interestingly seems to be a transcellular process in which TNFα and other pro-inflammatory interleukins, including IL-1β, IL-6, and IL-8, are sequentially secreted from neighboring cells, such as glia or macrophages [41, 196–198]. It remains uncertain whether the final COX increase and PG secretion occur mainly in neuronal or non-neuronal components. Neuronal COX expression was once reported to be elevated later than the initial pain induction by BK [199]. Exposing cultured rat trigeminal or DRG neurons to BK for 30 min to 3 h in evoked PGE_2_ release from the neurons, which was completely blocked by COX inhibitors in two independent studies [24, 43]. In both of those studies, B2 receptor activation appeared to be important to the secretory action.

#### PGE_2_-induced generation of neuropeptides and trophic factors

SP is a peptidergic neurotransmitter released from a subset of C-nociceptors and exacerbates inflammation and pain, as mentioned above [44]. The pro-inflammatory cytokine IL-1β has been shown to increase SP release by elevating COX-2 mRNA levels in rat DRG neurons [33]. Interestingly, NO facilitated IL-1β-induced COX-2 elevation in rat DRG neurons in a manner independent of its typical downstream messenger cyclic guanosine monophosphate (cGMP), and eventually facilitated SP release from these neurons [34]. The pharmacological antagonism of EP1 and EP2 receptors using AH-6809 showed no effect on PGE_2_-induced SP release from isolated rat renal somatosensory nerves, whereas the EP4 antagonists L-161982 and AH-23848 blocked it [200]. PGE_2_ not only contributes to SP release, but also promotes the expression of its receptor (SPR, also known as tachykinin receptor 1 or neurokinin 1 receptor) in cultured rat DRG neurons, and this elevated expression likely depends on the cAMP-PKA pathway [201]. The same study functionally demonstrated that the increase of intracellular Ca^2+^ in DRG neurons caused by SP exposure was significantly enhanced by PGE_2_ [201].

CGRP serves roles similar to those of SP in pain development [202]. PGE_2_-mediated cAMP signaling also positively regulates the release of CGRP [78]. In cultured DRG neurons, exposure to PGE_1_ or BK dose-dependently increased the expression and release of CGRP [42]. Pre-incubation with the COX inhibitor indomethacin suppressed BK-mediated CGRP release but not PGE_1_-induced CGRP release, indicating that COX-mediated PG production and possibly its autocrine and/or paracrine actions contribute to CGRP release from DRG neurons in BK-exposed conditions [42]. Morphine treatment, despite being known as a potent analgesic strategy, may exacerbate pain, such as when it is used as a preventive treatment for postoperative hyperalgesia [203]. Tumati and colleagues suggested a potential mechanism for this process by which sustained morphine treatment promotes PGE_2_-mediated CGRP release from somatosensory neurons [204].

Numerous studies have shown that up-regulated brain-derived neurotrophic factor (BDNF) in DRG neurons and the spinal cord contributes to the pathogenesis of chronic pain [205–209]. In a pSNL neuropathic pain model in rats, the injection of a COX-2 inhibitor (NS-398) or EP4 antagonist (AH23848) into the L4-L6 DRG lowered the injury-derived level of BDNF and improved mechanical hypersensitivity in a dose-dependent manner [35]. The same group replicated the paradigm by using explant cultures of the DRG, showing that a stabilized PGE_2_ analog, dimethyl PGE_2_ (dmPGE_2_), significantly elevated the BDNF level, dependent on EP1 and EP4 activation. Therefore, they suggested that nerve injury-derived PGE_2_ may facilitate BDNF production, contributing to neuropathic pain [35].

#### The effect of PGE_2_ on neuritogenesis

PGE_2_ plays a role in neurite outgrowth. Studies have elucidated PGE_2_-induced neurite elongation through EP2 or EP4 activation using neuronal cell lines, such as human neuroblastoma SK-N-BE(2) C cells, mouse neuroblastoma NG108–15 cells, somatosensory neuron-like ND7/23 cells, and motor neuron-like NSC-34 cells [210–213]. In addition, intraperitoneal injection of COX inhibitors, such as meloxicam and nimesulide, reduced adult neurogenesis in the hippocampus and the subventricular zone [30]. Indeed, treatment of cultured mouse DRG neurons with PGE_2_ has also been shown to promote neuritogenesis and axonal transport in an EP2 and cAMP-dependent manner [214]. It can be hypothesized that an upstream trophic factor could be using PG-signaling for this neuritogenesis, and vascular endothelial growth factor (VEGF) has been proposed as a candidate [215]. Cheng et al. demonstrated that VEGF stimulates COX-mediated production of PGE_2_ through the activation of one of its receptors, neuropilin-1 which is highly expressed in DRG neurons; they also showed that VEGF leads to the production of PGI_2_ [25]. VEGF-induced growth cone formation was abrogated by treatment with COX inhibitors, including indomethacin, SC-560 (for COX-1 inhibition), and NS-398 (for COX-2 inhibition). It remains unclear which EP receptors are critical and even which PGs are the best regulators for this process, because specific EP agonists were not successful in replicating this effect, whereas many endogenous prostaglandins were effective in rescuing growth cone collapse [25]. In a different study, however, the EP1/EP3 receptor agonist sulprostone was shown to cause retraction of the neurites of DRG neurons in a Rho-kinase-dependent fashion [108]. These morphologic theories await further investigations into how much the PG mechanisms contribute to pathological increases in nociceptor innervation and the following exacerbation of pain.

## PGI_2_

PGI_2_, also known as prostacyclin, is formed from PGH_2_ by the action of prostacyclin synthase (Fig. 1). It was first identified in vascular endothelial cells, where it causes vasodilation and inhibits platelet aggregation [216]. The evidence for the pro-nociceptive action of PGI_2_ has been accumulated as follows.

### PGI_2_ effects on nociceptive responses

Similar to the effects of PGE_2_, intradermal injection of PGI_2_ (1 μg) in rodent hind paws decreased the nociceptive threshold in response to mechanical stimuli, which was found to involve cAMP signaling in nociceptors [217]. The intraperitoneal injection of carbaprostacyclin (cPGI_2_), a stable prostacyclin analog, in sciatic nerve-transected rats increased the ectopic activity of DRG and dorsal horn neurons, suggesting that the generation of PGI_2_ might contribute to neuropathic pain [218]. Consistently, treatment with cicaprost, a PGI_2_ synthetic analog, has been shown to robustly increase cAMP production in rat adult DRG neurons to an even greater extent than PGE_2_ treatment [108]. That author hypothesized that the prostacyclin receptor might be responsible solely for elevating cAMP generation, whereas PGE_2_ might simultaneously activate multiple types of EPs, one of which uses the Gαi pathway and leads to a decrease in the cAMP level.

### The PGI_2_ receptor (IP) in nociceptors

One type of IP is conserved in mammals. mRNA transcripts for the IP-encoding gene *Ptgir* were readily detected in both small- and large-sized neurons in the L6 and S1 DRG of rodents [77, 219]. IP activation recruits the Gαs protein, activates adenylyl cyclase, and raises intracellular cAMP levels in a manner identical to that of the EP2, EP4, and DP1 receptors [220]. A genetic approach that generated mice deficient in *Ptgir* (which encodes IP protein) enriched the body of information about the roles of IP in pain and inflammation [221]. The intradermal injection of PGI_2_ enhanced BK-induced vascular permeability in wild-type mice, but not in *Ptgir*-deficient mice [221]. The *Ptgir*-ablated mice exhibited decreased edema formation when inflamed by carrageenan and reduced acetic acid-evoked writhing, compared with wild-type mice [221]. When PGI_2_ (2 μg) was injected intraperitoneally, 60% of the wild-types writhed, whereas the knockouts displayed no such nociceptive response [221]. Pharmacological approaches have shown consistent results. In DRG neurons, IP activation by its agonists such as cicaprost and iloprost heightened adenylyl cyclase activity [107, 219]. The cAMP accumulation caused by this IP activation led to the potentiation of capsaicin-, ATP-, and KCl-induced SP release in DRG neurons [219]. On the other hand, the application of an IP antagonist, 2-[4-(1H-indol-4-yloxymethyl)-benzyloxycarbonylamino]-3-phenyl-propionic acid, reversed the sensitized SP release [222]. More recently, Ng et al. have shown that the expression and downstream signaling cascade of IP were commonly conserved in somatosensory neurons and glial cells of DRG [113].

### TRPV1 potentiation by PGI_2_

As briefly mentioned in the PGE_2_ section above, PGI_2_ greatly potentiates TRPV1 activity. Pitchford et al. initially described the facilitation of TRPV1-mediated currents in DRG neurons upon PGI_2_ exposure, and knowledge about the details of signal transduction between IP activation and TRPV1 activation was enriched by Moriyama et al. [149, 151]. Briefly, IP activation by nanomolar amounts of PGI_2_ stimulates the Gαs-coupled adenylyl cyclase-PKA pathway in a relatively slow fashion (six minutes or longer), whereas micromolar concentrations of PGI_2_ additionally activate the Gαq-coupled PLC-PKC pathway on a fast time scale (~one minute) [151]. Both kinases phosphorylate TRPV1, leading to its heightened activity not only in response to its binding to ligands such as capsaicin, but also to heat and eventually contributing to TRPV1-mediated pain exacerbation.

## 15-Deoxy-Δ12,14-PGJ_2_ (15d-PGJ_2_)

PGD_2_ is further dehydrated into J series PGs, all of which contain a cyclopentenone ring [47]. The dehydration of PGD_2_ is a non-enzymatic process and consecutively produces PGJ_2_ (from the first dehydration) and 15d-PGJ_2_ (from the further dehydration and isomerization of PGJ_2_) (Fig. 1). The terminally dehydrated form 15d-PGJ_2_ can activate DP receptors, and also exert its actions by binding directly to other heterogeneous molecular targets such as the ion channel and nuclear receptor, as described below (Table 3).

**Table 3:** Functional effects of peripherally injected 15d-PGJ2 on nociceptive responses

Animal models	Injection	Dose	Effects	Remarks	References
Normal mouse	Intraplantar	32 nmol/25 μl	↑Licking, flinching	Disappeared in TRPA1^−/−^	[223]
Normal mouse	Intraplantar	15 nmol/20 μl	↑Licking, lifting	Disappeared in TRPA1^−/−^	[224]
Normal mouse	Intraplantar	15 nmol/10 μl	↑Licking, lifting	Disappeared in TRPA1^−/−^	[225]
Complete Freund’s adjuvant injected mouse	Intraplantar	1.5 or 15 mM/10 μl	↓ Mechanical hypersensitivity	Disappeared in TRPA1^−/−^	[226]
Sciatic nerve-injured rat	Intrathecal	50–200 μg/15 μl	↓ Mechanical and cold hypersensitivity	Attenuated by PPARγ antagonist	[227]
Sciatic nerve-injured rat	Intraperitoneal Iintracerebroventricular	100 μg	↔ Mechanical and cold hypersensitivity		[227]
Carrageenan-induced inflamed rat	Intraplantar	30–300 ng/100 μl	↓ Mechanical hypersensitivity	Attenuated by PPARγ antagonist	[228]
Formalin-induced TMJ rat	Into temporomandibular joint	100 ng/50 μl	↓ Mechanical hypersensitivity	Attenuated by PPARγ antagonist	[228]
Formalin-induced inflamed rat	Intraplantar	100 ng/50 μl	↔ Mechanical and cold hypersensitivity		[228]
PGE_2_-induced inflamed rat	Intraplantar	30–300 ng/50 μl	↓ Mechanical hypersensitivity	Attenuated by PPAR, Opioid receptor, Nitric oxide/cGMP/PKG)/K^+^_ATP_ pathway antagonists	[228]
PGE_2_-induced inflamed rat	Intraganglionic	100 ng/10 μl	↔ Mechanical and cold hypersensitivity		[228]
TNFα induced inflamed rat	Intraplantar	100 ng/100 μl	↓ Mechanical hypersensitivity		[228]
Formalin-induced TMJ rat	Into temporomandibular joint	1–100 ng/50 μl	↓ Mechanical hypersensitivity	Attenuated by PPARγ antagonist, Opioid receptor, (Nitric oxide/cGMP/PKG)/K^+^_ATP_ pathway antagonists	[229]

### TRPA1 activation and desensitization by 15d-PGJ_2_

Unlike TRPV1, for which most of the specific chemical activators mimic non-covalent capsaicin binding to the intracellular linker between the fourth and fifth transmembrane domains, the principle mode of ligand binding to TRPA1 is a covalent interaction [230–232]. When they can access several critical cysteine and/or lysine residues of the N-terminal cytoplasmic tail of the TRPA1 protein, electrophilic chemicals covalently bind to those residues, which eventually opens the channel pore.

15d-PGJ_2_, which has a highly reactive αβ-unsaturated carbonyl carbon, follows this covalent binding rule. In cultured DRG neurons, 20 μM 15d-PGJ_2_ raised intracellular Ca^2+^ levels in AITC-responsive (presumably TRPA1-positive) DRG neurons, which was undetectable in DRG neurons from Trpa1-deficient mice [223]. In both whole cell and inside-out patch clamp modes, 15d-PGJ_2_ evoked inward currents in TRPA1-overexpressing cells, suggesting that it activates TRPA1 in a membrane-delimited manner [223–225, 233, 234]. Two N-terminal cysteine residues (Cys421 and Cys621) of TRPA1 were determined to be the most critical for channel gating by 15d-PGJ_2_ [233]. On the other hand, 15d-PGJ_2_ is inert to other nociceptive TRPs such as TRPV1 and melastatin subtype 8 of TRP (TRPM8) [223, 225]. In a non-enzymatic manner similar to that in the production of the J series PGs, PGA_1_ and PGA_2_ are produced from PGE_2_ dehydration, and 8-iso-PGA_2_ comes from the dehydration of 8-iso-PGE_2_. These three dehydrated substances also contain an αβ-unsaturated carbonyl moiety in their cyclopentenone rings, and these electrophilic carbons can react with TRPA1 cysteines in the same covalent fashion, resulting in TRPA1 activation [224, 234].

Such in vitro reactivity was successfully extrapolated to the in vivo and circuit levels. The hind paw intraplantar administration of 15d-PGJ_2_ evoked nociceptive responses, including licking, biting, and flinching, in wild-type mice, whereas those responses were scarcely detected in Trpa1-deficient mice [223–225]. Cyclopentenone PGs robustly stimulated SP and CGRP release from nociceptors in the dorsal spinal cord and caused the expression of the c-fos gene, a marker of neuronal excitation, in dorsal spinal neurons [224].

Interestingly, this TRPA1-mediated mechanism also appears to be involved in the pain relief caused by 15d-PGJ_2_ treatment, which was once shown to reverse inflammatory pain in a CFA-injected animal model. Intraplantar application of 15d-PGJ_2_ to mouse hind paws reduced mechanical hypersensitivity in that model, but the effect was not observed in Trpa1-deficient mice [226]. The authors suggested that the analgesic effect may be due to subsequent desensitization of TRPA1 followed by 15d-PGJ_2_-induced TRPA1 activation [226]. The analgesic effect of 15d-PGJ_2_ and its potential molecular mechanisms have also been of recent interest in another context, as follows.

### Peroxisome proliferator-activated receptor γ (PPARγ) activation by 15d-PGJ_2_

When activated by their ligands, which are mostly lipid metabolites, the PPARs can alter multiple physiological functions, including glucose absorption, lipid balance, cell growth, and inflammation, by inducing transcriptional regulation [235]. Interestingly, PGDs and cyclopentenone PGs, such as 15d-PGJ_2_, PGJ_2_, PGA_1_, and PGA_2_ can activate PPARγ [236–238]. Among those examples, 15d-PGJ_2_ has been investigated in the context of pain modulation. Intraplantar injection of 15d-PGJ_2_ has been shown to diminish the mechanical hypersensitivity of rat hind paws inflamed by carrageenan or PGE_2_ [228]. This effect was reduced by treatment with the PPARγ antagonist, GW9662 [228]. Interestingly, intra-DRG injection of 15d-PGJ_2_ did not successfully relieve this mechanical hypersensitivity [228]. The same group also used formalin- and serotonin-induced temporomandibular joint (TMJ) pain and demonstrated that administration of 15d-PGJ_2_ into the TMJ suppressed hyper-nociception [228, 229]. The authors’ pharmacological explorations of the downstream mechanisms of this 15d-PGJ_2_ signaling suggested that non-neuronal cells, such as macrophages, and κ and δ opioid receptors could contribute to its analgesic mechanisms.

In a different study, Churi and colleagues demonstrated the presence of PPARγ mRNA and proteins in the dorsal horn of the rat spinal cord [227]. The intrathecal injection of endogenous and synthetic PPARγ ligands (15d-PGJ_2_ or rosiglitazone) dose-dependently alleviated the mechanical and cold hypersensitivity of rats with sciatic nerve injury [227]. The analgesic effects of 15d-PGJ_2_ and rosiglitazone were blunted by the co-administration of a PPARγ antagonist (bisphenol A diglycidyl ether, also known as BADGE). In the same study, intraperitoneal and intracerebroventricular injections of PPARγ agonists failed to reduce mechanical and cold hypersensitivities [227]. Future studies will quantitatively clarify whether TRPA1 activation-mediated pain generation, TRPA1 desensitization-mediated pain reduction, or PPARγ-mediated pain reduction pre-dominates in physiological and pathological states. It also must be determined whether 15d-PGJ_2_ displays differential effects on the peripheral pain pathway depending on its location and concentration.

## Thromboxane A_2_ (TXA_2_)

TXA_2_, generated by TXA_2_ synthase 1 (TBXAS1) directly from PGH_2_, is an unstable prostanoid with a chemical half-life of about 30 s and is further spontaneously converted into TXB_2_, an inactive metabolite [239, 240] (Fig. 1). TXA_2_ activates its specific Gαq-protein coupled receptor TP (thromboxane receptor), which initiates the PLC signal transduction pathway [241] (Hirata et al., 1991). TXA_2_ has been intensely investigated with regard to its function in platelet aggregation and smooth muscle contraction [242–244]. Recent TXA_2_ studies have expanded into its roles in other physiological and pathological circumstances such as cancer metastasis, immune responses, and asthma (See review: [244]). Several studies have also looked into its role in visceral sensory and somatosensory contexts.

### Nociceptor excitation by TXA_2_ mimetics

Due to the extremely short half-life of TXA_2_, the TXA_2_ mimetic U46619 is often used to study the roles of TXA_2_ in sensory nerve-mediated responses. The infusion of U46619 into vagal C-fibers innervating the lung elicited massive firing of those nerves [245, 246]. This excitation may help explain how TXA_2_ caused the reflexive pulmonary hypertension and rapid shallow breathing observed in previous studies and indicates that vagal nerve interaction with TXA_2_ may importantly contribute to cardiorespiratory feedback regulation [245–247]. Since that study, diverse measures from different animal models have confirmed this U46619 response led by the excitation of autonomic C-fibers, such as those associated with the vagal reflex-mediated knee-jerk reflex of cats, tachypnea and bradycardia of rabbits, and chemoreceptor activation in rats [248–251]. U46619 could also stimulate somatosensory nociceptors. Systemic injection of U46619 through the abdominal aorta of cats led to increases in the baseline impulses of action potential generated from a subset of Aδ-fibers (~ 30%) and C-fibers (~ 60%) both of which innervate the hind limbs [252–254].

The use of a TP receptor antagonist may help determine the actions of endogenous TXA_2_ and it has also been tried in the vagal components [255]. Treatment with the selective TP antagonist BM13177 attenuated the increased activity of cardiac sensory afferents in ischemic cats [255]. The injection of U46619 into the left atrium further excited cardiac afferents in a dose-dependent manner, which was suppressed by treatment with BM13177 or the inhibitor, PKC-(19–36). Therefore, these findings suggest that endogenous TXA_2_ generated in ischemic conditions may stimulate cardiac sensory afferents through a TP-mediated PLC-PKC signal transduction pathway (Fig. 3).

### TP expression in nociceptors

*Tbxa2r* mRNA transcripts for TP expression were detected in a single cell RT-PCR analysis of neurons cultured from nodose ganglia (which contain the cell bodies of vagal afferents) and thoracic DRG excised from adult rabbits [256, 257]. The TP-positive population was slightly larger in the nodose neurons (~ 18%) than in the DRG neurons (~ 12%), and more than half of those neurons were also TRPV1-positive [257]. Using immunohistochemistry in mice, Andoh and colleagues showed that not only the small-to-medium-sized DRG neurons that innervate the skin, but also epidermal keratinocytes express TPs [258]. Whereas most keratinocytes expressed both TBXAS1 and TP, 78% of small-sized neurons, 48% of medium-sized neurons, and 13% of large-diameter neurons exhibited TP-immunoreactivity while being negative for TBXAS1, which indicates that TXA_2_ is generated mostly from keratinocytes and may interact with TP in keratinocytes and nociceptors in autocrine and paracrine manners [258]. Those authors looked further into that paradigm in the context of itchiness. The intradermal injection of U46619 caused scratching behavior in ICR mice, which was attenuated by treatment with the TP receptor antagonist ONO-3708 [258]. Such U46619-induced scratching behaviors were almost absent in the TP receptor-deficient mice [258]. In cultured mouse DRG neurons and keratinocytes, Andoh and colleagues confirmed that U46619 can induce increases in intracellular Ca^2+^ through TP receptor signaling, which possibly indicates that Gαq-PLC-mediated Ca^2+^ mobilization contributes to the phenotype [258]. These findings suggest that TP activation by TXA_2_ involving keratinocytes and DRG neurons may compose an itch-induction mechanism.

## Limitations in the current knowledge about PGs

Despite the details that have accumulated about the pro-nociceptive mechanisms of PGs, those still remain to be further systematized and extended. Although there has already been a clinically available series of PG-modulable analgesic strategies, they are not now considered quite successful, particularly in treating neuropathic pain [259]. This situation requires us to consider how immature the current knowledge is and what remains to be solved. For example, a more systematic view likely needs to be constructed, embracing the diverse and newly suggested participant molecules in PG-associated cellular processes and quantifying their contributions to final pain outcomes. In addition, a circuit-specific inflammatory etiology involving a multitude of PGs may need to be monitored precisely because functional plasticity in the somatosensory circuit is likely a central cause of chronic pain. More information may also be required on how different PG contributions to pain severity vary chronologically during its exacerbation, since some PGs might be more important at the onset of the plastic changes in the pain circuit and others at the maintenance stage. The effects of some clinically available COX inhibitors have been highlighted in neuropathic animal models as mentioned above. However, considering the high doses used in most of those studies, their efficacy and potency do not seem to be realized for the human clinical setting. Possibly more potent but, to avoid adverse effects in other tissues, highly specific strategies to the somatosensory circuit may have next opportunities. It may also implicate the potential diversity of human pain mechanisms that can hardly be covered with a limited number of the current animal models. More fundamentally, considering the possible species differences, a reappraisal of the current models, the development of newly optimized ones on the mechanistic basis, and the standardization of humanized platforms may be required [260]. Collectively, overcoming such existing inadequacies may could enable new possibilities for clinical applications.

COX produces not only PGs but also resolvent lipids such as resolvins, maresin, and neuroprotectins [261, 262]. The resolvent lipids promote the termination of inflammation by regulating macrophages, monocytes, and microglia, and they also directly mitigate nociceptor excitability. Thus, the outcomes of their possible reduction by COX inhibition may need to be taken into account. In this regard, further downstream targeting is likely to be more effective in the peripheral somatosensory system, while causing less disturbance to the endogenous resolution mechanism by, for example, selectively blocking pro-nociceptive EP2, EP4, DP1, and IP receptors or selectively activating EP3 and DP2 in nociceptors. Such receptor specific-protocols are becoming popular in various biomedical fields, including not only pain-related diseases, but also cancer and asthma [263, 264]. Quantitative information about which PGs and receptors cause the predominant outcomes can help in filtering more appropriate strategies. For example, DP2 activation can even reverse the EP-mediated excitation of DRG neurons [265]. On the other hand, DP2 was once shown to contribute relatively less predominantly than DP1 to modifying TTX-R activity compared to DP1, as mentioned above [115]. Despite not being limited to the status of nociceptor excitability, but reflecting their central contributions together, EP and DP knockouts were compared in a pain study, which indicated that EP4 activation may be more important in heat pain, EP1and 3 seem to be involved in heat pain reduction, and EP2 and DP1 may contribute to formalin-induced pain [266].

Understanding farther downstream signaling might also be informative in building therapeutic hypotheses. As shown in the reciprocal relationship between PGs and BK, certain merge points present within a multitude of signaling cascades that are initiated by different but comparably important inflammatory pain mediators can be hypothesized as bottlenecks to gain a heightened nociception. Intracellular Ca^2+^, a common intracellular messenger in PGE_2_ and BK-induced excitation, has also been shown to be important in the potentiation of the trigeminal PGE_2_/BK response through Y2 neuropeptide Y receptor activation [267]. Histamine-induced rat DRG excitation is potentiated by PGE_2_ [268]. An influx of extracellular Ca^2+^ was raised as a key mechanism for the initiation of this potentiation [268], but for the Ca^2+^ carrying targets involved in this mobilization, we currently know only the molecules with less tissue specificity. Because this limited state of knowledge about downstream signaling makes therapeutic translation stay immature, future exploration is needed to create a comprehensive list of nociceptor-specific candidates for merger contributors.

The processes leading to PG breakdown have not been well elucidated in pain research investigating PG-associated mechanisms. The balance between the rates of PG production and degradation might affect pain states, because it determines whether the concentration of PGs reaches an effective window for inducing receptor activation and pain signals. Two enzymes that directly metabolize PGE_2_ and TXB_2_, respectively, have been shown: 15-hydroxyprostaglandin dehydrogenase (15-PGDH also known as HPGD) and 11-hydroxythromboxane B_2_ dehydrogenase (11-TXB2DH) [269]. Because TXA_2_ is the thromboxane most important in pro-nociception but is chemically labile, PG-degrading enzymes might be more critical in pain modulation than thromboxane-degrading ones. It can be hypothesized that enhancement of 15-PGDH could decrease the concentration of PG, helping to normalize nociceptor excitability. Inhibition of the same enzyme could cause an opposite result. Currently, only one inhibitor is available, and it has recently been studied in the regeneration field. The inhibitor SW033291 has been demonstrated to effectively raise tissue-wide levels of PG and ultimately contribute to regeneration [270]. It might be interesting to explore how it affects inflammation and pain states in the nervous system.

## Perspectives and conclusion

This review has highlighted the roles of arachidonic acid-derived PGs in nociception relayed by the peripheral somatosensory system. The current enlargement of knowledge may promote the design of a variety of anti-nociceptive strategies by surgically modulating specific PG actions. Such achievements may help to overcome the comparatively narrow focus and reliance on COX inhibition for pain control. Because a number of steps in the PG-mediated pain induction mechanisms remain less studied, their clarification may need to precede devising therapeutic tools targeting PGs in nociceptors. Pain diseases are diverse. The modulation of a PG-related molecule must be mechanistically assessed to determine which severity, chronicity, inflammatory phase, and phenotype is more sensitive to it. Trigeminal mechanisms that, while not fully introduced here, operate similarly to the peripheral somatosensory circuit, appear to crucially use PG-mediated signaling in headaches, migraine pain, and meningeal neurogenic inflammation [271]. Recent progress in that field could be useful in determining therapeutic directions [68, 272–276]. The PGs listed here are only the primary products enzymatically processed from the arachidonic acid-PGH_2_ axis. The effects on nociceptors of non-enzymatic products, including 12-hydroxyheptadecatrienoic acid, further metabolized substances like PGF_1α_, or other polyunsaturated fatty acid-derived PGs outside that axis also need to be examined in the future and compared with those described above. Such future efforts will provide a full picture of the mechanisms underlying somatosensory excitation and widen the pool for painkilling targets.

## Data Availability

Not applicable.

## Abbreviations

15-PGDH: 15-hydroxyprostaglandin dehydrogenase
AHP_Slow_: Slow spike after-hyperpolarization
AITC: Allyl isothiocyanate
AKAP150: A-kinase anchoring protein 150
BDNF: Brain-derived neurotrophic factor
BK: Bradykinin
CCI: Chronic constriction injury
CFA: Complete Freund’s adjuvant
CGRP: Calcitonin gene-related peptide
COXs: Cyclooxygenases
DRG: Dorsal root ganglia
HCN: Hyperpolarization-activated cyclic nucleotide gated channel
H-PGDS: Hematopoietic PGD synthase
IB4: Isolectin B4
Ih: Hyperpolarization-activated cation current
K_Ca_: Ca^2+^-activated K^+^ channel
KCC: K-Cl cotransporter
L-NMMA: N^G^-monomethyl-L-arginine
L-PGDS: Lipocalin-type PGD synthase
LPS: Lipopolysaccharide
mPGES-1: Microsomal PGES-1
NGF: Nerve growth factor
NKCC: Na-K-Cl cotransporter 1
ODN: Oligodeoxynucleotide
PGES: PGE_2_ synthase
PGs: Prostaglandins
PPARγ: Peroxisome proliferator-activated receptor γ
pSNL: Partial sciatic nerve ligation
RT-PCR: Reverse transcription-polymerase chain reaction
SP: Substance P
SPR: Substance P receptor
TBXAS1: TXA_2_ synthase 1
TMJ: Temporomandibular joint
TNF: Tumor necrosis factor
TRP: Transient receptor potential
TTX: Tetrodotoxin
TTX-R: Tetrodotoxin-resistant voltage-gated Na^+^ channel
TTX-S: Tetrodotoxin-sensitive voltage-gated Na^+^ channel
TX: Thromboxane
VEGF: Vascular endothelial growth factor
